# Virtual screening of Indonesian herbal compounds as COVID-19 supportive therapy: machine learning and pharmacophore modeling approaches

**DOI:** 10.1186/s12906-022-03686-y

**Published:** 2022-08-03

**Authors:** Linda Erlina, Rafika Indah Paramita, Wisnu Ananta Kusuma, Fadilah Fadilah, Aryo Tedjo, Irandi Putra Pratomo, Nabila Sekar Ramadhanti, Ahmad Kamal Nasution, Fadhlal Khaliq Surado, Aries Fitriawan, Khaerunissa Anbar Istiadi, Arry Yanuar

**Affiliations:** 1 Department of Medical Chemistry, Faculty of Medicine, Universitas Indonesia, Jalan Salemba Raya number 4, Jakarta, 10430 Indonesia; 2 Bioinformatics Core Facilities - IMERI, Faculty of Medicine, Universitas Indonesia, Jalan Salemba Raya number 6, Jakarta, 10430 Indonesia; 3 Department of Computer Science, Faculty of Mathematics and Natural Science, IPB University, Jalan Meranti Wing 20 level 5 Kampus IPB, Bogor, West Java 16680 Indonesia; 4 Tropical Biopharmaca Research Center, Institute of Research and Community Empowerment, IPB University, Jalan Taman Kencana number 3, Bogor, West Java 16128 Indonesia; 5 Department of Pulmonology and Respiratory Medicine, Faculty of Medicine, Universitas Indonesia – Universitas Indonesia Hospital, Depok, West Java 16424 Indonesia; 6 Department of Biology, Institut Teknologi Sumatera, Bandar Lampung, Lampung 35365 Indonesia; 7 Biomedical Computational and Drug Design Laboratory, Faculty of Pharmacy, Universitas Indonesia, Kampus Baru UI, Depok, West Java 16424 Indonesia

**Keywords:** COVID-19, Machine Learning, Pharmacophore Modeling, Molecular Docking, Indonesian Herbal Compounds, 3CLPro, SARS-CoV-2

## Abstract

**Background:**

The number of COVID-19 cases continues to grow in Indonesia. This phenomenon motivates researchers to find alternative drugs that function for prevention or treatment. Due to the rich biodiversity of Indonesian medicinal plants, one alternative is to examine the potential of herbal medicines to support COVID therapy. This study aims to identify potential compound candidates in Indonesian herbal using a machine learning and pharmacophore modeling approaches.

**Methods:**

We used three classification methods that had different decision-making processes: support vector machine (SVM), multilayer perceptron (MLP), and random forest (RF). For the pharmacophore modeling approach, we performed a structure-based analysis on the 3D structure of the main protease SARS-CoV-2 (3CLPro) and repurposed SARS, MERS, and SARS-CoV-2 drugs identified from the literature as datasets in the ligand-based method. Lastly, we used molecular docking to analyze the interactions between the 3CLpro and 14 hit compounds from the Indonesian Herbal Database (HerbalDB), with lopinavir as a positive control.

**Results:**

From the molecular docking analysis, we found six potential compounds that may act as the main proteases of the SARS-CoV-2 inhibitor: hesperidin, kaempferol-3,4'-di-O-methyl ether (Ermanin); myricetin-3-glucoside, peonidin 3-(4’-arabinosylglucoside); quercetin 3-(2G-rhamnosylrutinoside); and rhamnetin 3-mannosyl-(1-2)-alloside.

**Conclusions:**

Our layered virtual screening with machine learning and pharmacophore modeling approaches provided a more objective and optimal virtual screening and avoided subjective decision making of the results. Herbal compounds from the screening, i.e. hesperidin, kaempferol-3,4'-di-O-methyl ether (Ermanin); myricetin-3-glucoside, peonidin 3-(4’-arabinosylglucoside); quercetin 3-(2G-rhamnosylrutinoside); and rhamnetin 3-mannosyl-(1-2)-alloside are potential antiviral candidates for SARS-CoV-2. *Moringa oleifera* and *Psidium guajava* that consist of those compounds, could be an alternative option as COVID-19 herbal preventions.

**Supplementary Information:**

The online version contains supplementary material available at 10.1186/s12906-022-03686-y.

## Background

The new coronavirus, severe acute respiratory syndrome coronavirus 2 (SARS-CoV-2), was first identified in Wuhan, China, in December 2019 [1]. SARS-CoV-2 belongs to the Coronaviridae family, a single-stranded RNA virus that is widespread among humans and other mammals, causing a wide range of infections from common cold symptoms to fatal illnesses, such as severe respiratory syndrome [2, 3]. The latest spread of coronavirus disease 2019 (COVID-19) caused by SARS-CoV-2 in Indonesia has reached 6,048,685 cases, with 156,396 mortalities as of May 10^th^, 2022 (https://covid19.go.id). Unfortunately, these numbers continue to increase, and the effective drugs are still on discoveries.

There are two categories of anti-coronavirus therapy depending on the target: one acts on the human immune system or human cells, and the other acts on the coronavirus itself. The human innate immune system response plays an essential role in controlling the replication and infection of coronavirus and in enhancing the immune response [4]. Blocking the signaling pathways of human cells required for virus replication may exhibit a specific antiviral effect. The therapies that work on the coronavirus itself include preventing the synthesis of viral RNA by acting on the genetic material of the virus, inhibiting virus replication through blocking critical enzymes of the virus, blocking the virus from binding to human cell receptors, or inhibiting the viral assembly process by modulating several structural proteins [5].

Exploring new medicines for emerging and rapidly spreading diseases, such as COVID-19, may be performed through a drug repurposing strategy to bypass the pre-clinical steps that usually require laborious work and resources [6]. Drug repurposing is conducted by finding new uses for already registered drug compounds. Drug repurposing can be typically performed by analyzing the interaction of compounds drugs with proteins related to the diseases (drug-target interaction or DTI) and subsequently predicting new DTIs in which the interactions are previously unknown [7, 8]. Drug repurposing is commonly conducted in conventional medicines. However, in Indonesia, where people are more familiar with using herbal to care for their health in daily life, there is a need to consider developing anti-viral agents from well-known herbs, which people may easily use.

In drug discovery, a drug repurposing strategy could decrease the time of 2-14 years of the process. To support the drug repurposing strategy and help reduce the time and cost of laboratory experiments, we used virtual screening [9]. The virtual screening process typically identifies the potential binding of structures to each other, for instance, a drug compound and its protein targets. Virtual screening is based on compound similarity or database docking [10]. However, cheminformatic studies have found that computer science approaches, such as pharmacophore analysis [9] and some machine learning techniques, help identify the interaction between a drug and its protein targets [10–12]. This study used layered virtual screening by combining machine learning model prediction and pharmacophore modelling approaches.

Fitriawan *et al.* [10] developed a deep learning classification model for a nicotinamide adenine dinucleotide (NAD) protein target problem and used PubChem fingerprints as a feature. Dhanda *et al.* [11] used a combination of hybrid fingerprint models to develop a support vector machine (SVM) prediction for drug compounds. Liu [12] used different approaches to combine the classifier, called ensemble machine learning. Johnson and Maggiora [13] analyzed chemical compound similarity and found that compounds with similar structures have similar properties. Based on this concept, adding a machine learning method could improve the performance in finding drug compounds.

In this study, we aimed to find potential candidate compounds for anti-SARS-CoV-2 therapy in Indonesian plants, with the primary objective of preventing infection. We curated these candidates using big data analysis and machine learning and compared the results with those obtained from a pharmacophore modeling approach. Consensus candidate compounds and proteins from both approaches were validated using molecular docking. The results of this study produced several potential compound candidates that could be targeted for preventing viral infection, as the candidate plants (especially commodity crops) may be used easily and directly by the community.

## Methods

**Fig. 1:**
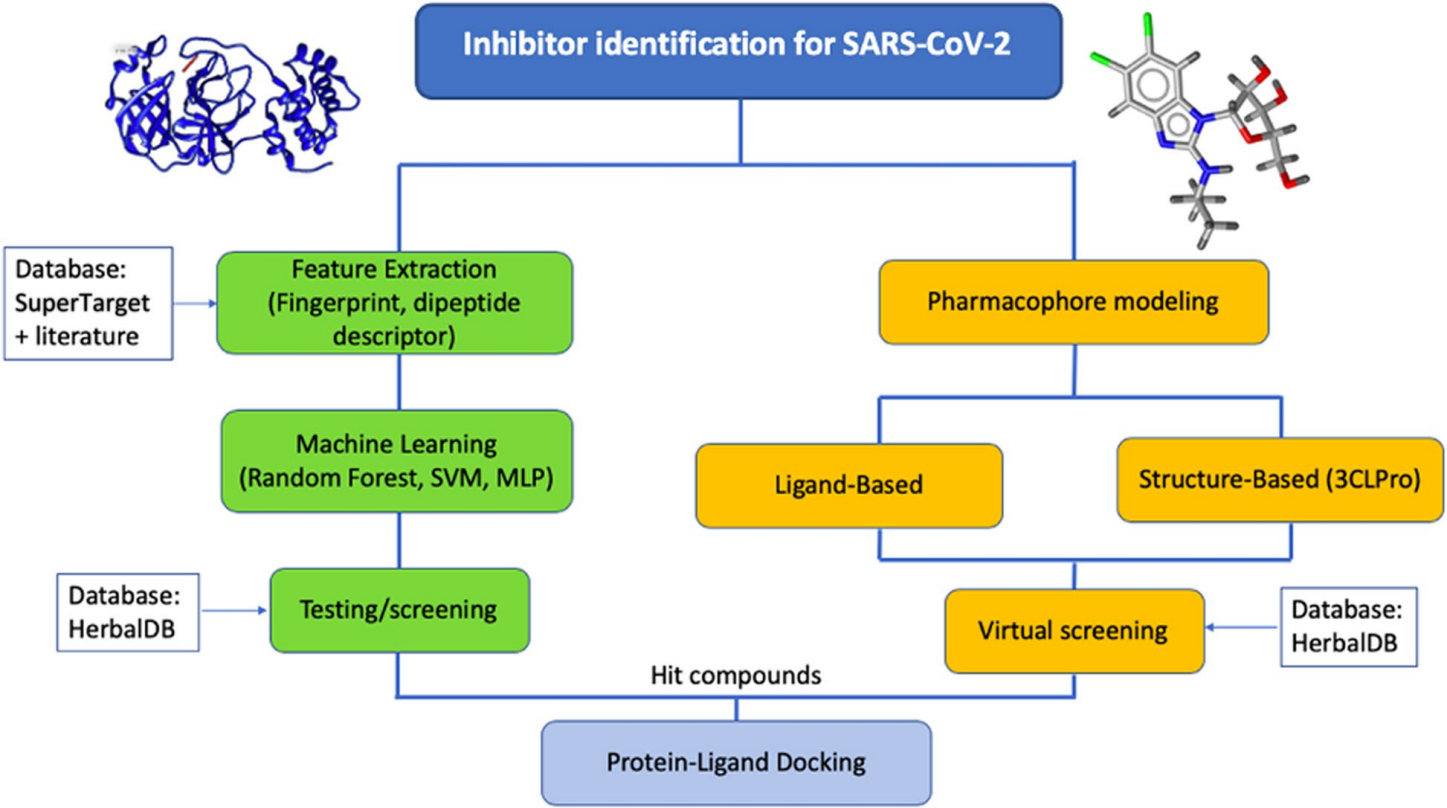
Study Workflow

In this study, we combined two approaches to screen for candidate drugs: machine learning and pharmacophore modeling. The compounds that overlap from the two approaches were further analyzed using molecular docking. The graphical method in this study is represented in Fig. 1.

### Machine learning approach

There were four steps in the DTI prediction using machine learning approach. This process started with a literature review to identify drugs and protein target interactions (DTI) from public research studies. DTI data extracted from literature and taken from some public domain databases were used as the training dataset. The chemical structure and genomic sequence features were then extracted from the identified drugs and protein targets. Next, the training datasets were tuned to obtain the hyperparameters, which were used to generate the optimal model. The last step was to utilize the predictive models to make predictions for the herbal compounds’ dataset. The machine-learning approach was conducted on Intel (R) Xeon (R) Silver 4110 CPU @ 2.10GHz, with 65.58 GB memory. All data and source codes of the machine learning approach used in this research can be accessed at https://github.com/TropBRC-BioinfoLab/virtual-screening-covid19.

### Data acquisition

The original datasets used in this study, which consisted of drugs and protein targets, were obtained from Li and Clercq [6] and Wu et al. [5] in 2020. There were 81 virus-based drugs (Additional file 1), 17 human-based drugs (Additional file 2), 15 host-based proteins, and eight virus-based proteins (Table 1). Wu et al. [5] systematically analyzed proteins encoded by the SARS-CoV-2 gene, compared them to the target proteins from other coronaviruses, and predicted their structure using homology modeling. Li and Clercq [6] investigated the potential for reusing antiviral agents based on the therapeutic experience with two infections caused by other coronaviruses. The antiviral drugs’ potential in [5, 6] was determined by a significant binding affinity score on drug-target interaction. To extend the exploration of drug-target interactions, we input protein targets and drugs into SuperTarget web resources (bioinf-apache.charite.de/supertarget) [19]. The outputs of SuperTarget were not only the interactions between drugs and protein targets but also the new protein targets and new drugs (Table 2) that were not previously mentioned in [5, 6]*.* The total number of data obtained from the literature and SuperTarget was 119 drugs, 335 protein targets, and 685 interactions (Additional file 3). The total possible interaction that might exist was 119 drugs*335 targets = 39,865 interactions. Thus, the total dataset had 39,865 samples that consisted of 685 samples with positive interactions and 39,180 samples with unknown interactions (negative).

**Table 1:** List of potential protein target related to COVID-19

Virus-based protein			Host-based protein			
PDB/Uniprot ID	Protein	Reference	Uniprot name	Uniprot ID	Protein	Reference
6LU7:A	3CLPro	[5]	ACE2	Q9BYF1	ACE2	[14]
PLpro_SARS-CoV-2	PLPro	[5]	AKT1	P31749	AKT	[15]
K4LC41						
			PYRD	Q02127	DHODH	[16]
yp_009725307.1	RdRp	[5]	PPIA	P62937		
			PPIG	Q13427		
6M0J:E	Spike-ACE2	[5]	FKBP5	Q13451		
6LZG:A			FKBP4	Q02790		
6VSB			FKBP2	P26885		
6M0J:A						
			CYP5	P52013	PPIASE	[17]
			FKB1B	P68106		
			PPIB	P23284		
			PPIC	P45877		
			PPIH	O43447		
			FKB1A	P62942		
			IL6RB	P40189	IL-6	[18]

**Table 2:** List of potential drug explored from SuperTarget database

Drug	Protein Target
Moexipril hydrochloride	ACE2
Arsentrioxide	AKT1
Arthrocine	
Celecoxib	
Erlotinib	
Gefitinib	
Imatinib Mesylate	
Lapatinib ditosylate	
Simvastatin	
Sorafenibum	
Sunitinib	
Atovaquone	PYRD, PPIA, PPIG
Essigsaeure	
Huanghuahaosu	
Hydroxycinchophene	
Leflunomide	
Rapamycin	FKBP5, FKBP4, FKBP2, FKB1B, FKB1A
Athylenglykol	FKBP4
Methylsulfinylmethane	
Dithiothreitol	CYP5_CAEEL
Carboxypyrrolidine	PPIB, PPIC, PPIH
Pimecrolimus	FKB1A
Tacrolimus	
Thiabendazole	

As described earlier, this study aimed to identify the potential compounds in Indonesian plants as anti-SARS-CoV-2 therapy with the primary objective of preventing infection. Thus, we collected 400 Indonesian herbal compounds obtained from HerbalDB (herbaldb.farmasi.ui.ac.id) [20] as a testing dataset. This dataset had no label. Our proposed model predicted the labels as positive or negative.

### Drug-target representation

In DTI prediction, the input data required numerical representations of compounds and proteins on the classification model. The compound descriptors are the simplified molecular-input line-entry system, which may be used to effectively obtain the fingerprint of a chemical structure. Fingerprint is the encoding of a compound into a Boolean fingerprint vector representing the existence of a substructure within the compound's molecule. PubChem [21] issued 881 structural keys. The structural key was used as a compound similarity measure for similar compounds found on their website http://pubchem.ncbi.nlm.nih.gov.

PubChem fingerprint was chosen because it has the ability to explain more characteristics of a compound. PubChem fingerprint consisted of 881 0/1 features. This indicates that this characterization only needs one bit of storage for every feature in a compound, whereas using other kinds of features involves a floating point number might need up to 32 bits for one feature. This small fingerprint helps to accelerate the machine learning process. PubChem fingerprint uses a substructure key based on the 2D structure of a compound that is also used for similarity search [22], the same as the purpose of this paper, which explored herbal compounds with similar features from existing compound-protein interactions. Another study about database fingerprint (DFP), which includes PubChem fingerprint, suggested that DFP is enough for compound dataset representation [23].

The simplest of protein descriptors is amino acid composition. There are 20 components, each of which is represented using a single letter code. However, the weakness of amino acid composition descriptors is that the same amino acid composition may correspond to diverse sequences as sequence order is lost [24]. The dipeptide composition (DC) can cover the sequence order information. Thus, this study used DC as a protein descriptor. Dipeptides are combinations of two amino acid components (such as AA, AR, AN, AD, AC). DC converts protein sequences into 400 features. DC can be defined by Eq. (1).

1 $${X}_{dep(i)}=\frac{n_{dep(i)}}{N}$$

where *dep(i)* is the *i*-th dipeptide of 400 dipeptides, *X*_*dep(i)*_ represents the ratio of occurrences of *dep(i)*, *n*_*dep(i)*_ is the number of occurrences of *dep(i)*, and *N* is the sum of occurrences of all dipeptides.

The reason for using DC is that it is easily extracted from protein sequences, consists of 400 features that cover characteristics of a protein, and can obtain good performance in the problem of classification or prediction [25]. Ong et al. [26] comparatively evaluated the effectiveness of the protein descriptor sets using the same machine learning method and parameter optimization algorithm and examined whether the combination of descriptors improved the predictive performance. In the study [26], the authors used six individual descriptor sets (Amino acid composition, dipeptide composition, normalized Moreau – Broto autocorrelation, Moran autocorrelation, Geary autocorrelation, and descriptors of composition, transition and distribution), and four combination sets (combination of sequence composition and correlation of physicochemical, combination of sequence composition and square correlation of physicochemical, combination of sequence compositions, and combination of all sets). The results showed that all descriptors used in the study generally obtained good and similar performance. Moreover, the use of combination descriptor sets provided only slightly better prediction than the use of individual descriptor sets.

In this research, PubChem fingerprint and dipeptide descriptor were used as the drug compound features and the protein target features, respectively. PubChem fingerprint was acquired using the PubChemPy library in Python, while the dipeptide descriptor was calculated using the *protr* package in R. Each record consisted of 881 compound fingerprints and 400 protein dipeptide descriptors. A total of 1281 features represented the DTI samples.

### Machine learning methods

**Fig. 2:**
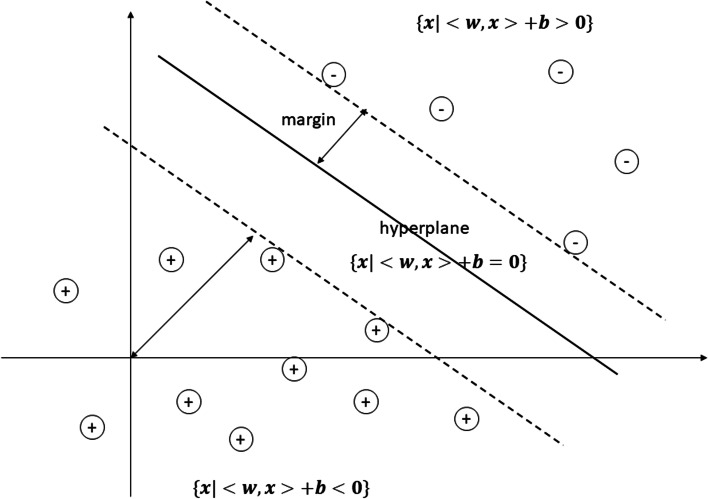
Support vector machine method (*x* is the data, *w* is the weight vector, *b* is the bias score, *ε* is the minimum error) [28]

We used three machine learning methods that had different ways of deciding to build a model for classifying objects into the appropriate class in the binary classification problem. The SVM makes a decision based on hyperplane [27] (Fig. 2). The hyperplane is obtained by minimizing the maximum distance of the hyperplane and support vector (margins) with a minimum error that can be calculated based on the following equation:

2 $$P\left(w,b\right)=\frac{1}{2}{\left\Vert w\right\Vert}^2+\varepsilon$$

with *w* as weight vector, *b* as bias score, and *ε* as a minimum error from the calculation [29]

To avoid misclassification of each training sample, the Regularization parameter (C parameter) is introduced to optimize the margin. Eq. 2 can be improved as follows [30]:

3 $${\mathit{\min}}_{w,b,\varepsilon}\frac{1}{2}{w}^Tw+C{\sum}_{i=1}^l{\varepsilon}_i,$$

$$\mathrm{subject}\ \mathrm{to}\ {y}_i\left({w}^T\phi \left({x}_i\right)+b\right)\ge 1-{\varepsilon}_i,$$

$${\varepsilon}_i\ge 0,i=1,\dots, l,$$

where *ϕ*(*x*_*i*_) maps *x*_*i*_ into a higher dimensional space and C > 0 is the regularization parameter. The problem in Eq. 3 considers high dimensional data. Due to the possible high dimensionality of the vector variable w, solve the following dual problem [30]:

Using the primal-dual relationship, the optimal *w* is

4 $$w={\sum}_{i=1}^l{y}_i{\alpha}_i\phi \left({x}_i\right),$$

and the decision function is

5 $$\mathit{\operatorname{sgn}}\left({w}^T\phi (x)+b\right)=\mathit{\operatorname{sgn}}\left({\sum}_{i=1}^l{y}_i{\alpha}_i\ K\left({x}_i,x\right)+b\right),$$

The fitting model was obtained by tuning the regularization parameter and the parameter of the kernel (K) used in training. In this research, we used the RBF kernel, which is defined as [31]:

6 $$K\left({x}_i,x\right)=\mathit{\exp}\left(-\gamma {\left\Vert {x}_i-x\right\Vert}^2\right),$$

where *x* is the data, *i* is the dimension and *γ* is a free parameter. The *γ* parameter from Eq. 6 and regularization parameter C from Eq. 3 needed to be decided before training the data. To find the best score for the parameter (C, *γ*), we used the grid search with 5-cross validation [29].

The second machine learning method used in this study was Random Forest (RF). RF is a bagging-type ensemble of uncorrelated decision trees that trains several trees in parallel and uses voting or the majority decision of the trees as the final decision [32]. RF classifier filters the attribute of the data by using Gini Index, as defined in [33]:

7 $$\sum\sum\limits_{j\ne i}\left(f\left({C}_i,T\right)/\left|T\right|\right)\left(f\left({C}_{j},T\right)/\left|T\right|\right),$$

where *T* is a training set, and a random data in class *C*_*j*_, and (*f*(*C*_*i*_, *T*))/|*T*| is a probability of where the data belongs in class *C*_*i*_. Random forest (RF) constructed many decision trees based on averaging random selection of predictor variables. When constructing the trees, whenever a split was considered, a random selection of *m* predictors was selected as a subset of split candidates from the complete set of predictors. The fitting model can be obtained by tuning hyperparameters. The important hyperparameters included the number of subsamples of the original features used to build each decision tree (*mtry*) and the weight assigned to each class. We used a grid search with 5-cross-validation when conducting tuning hyperparameter optimization to ensure that the random forest was exposed to all the statistical distributions in the training dataset.

The third machine learning method used in this research was multilayer perceptron (MLP). MLP works based on an artificial neural network [34]. In the MLP, the input was first transformed using a non-linear transformation. The input nodes in the input layer provided information from the outside to the network, for instance *m* is a number of dimensions for input and the set of features are consisted of a neurons set {*x*_*i*_|*x*_1_, *x*_2_, …, *x*_*m*_}. The hidden layer nodes performed computations and transferred information from the input nodes to the output nodes, it transform the values from the previous layer with weight *w*_1_*x*_1_ + *w*_2_*x*_2_ + … + *w*_*m*_*x*_*m*_, and a non-linear activation function *g*(.) : *R* → *R*- [35]. An MLP can have one or more hidden layers. In this research, we used two hidden layers. Lastly, the output nodes were responsible for computation and transferring information from the network to the outside. The optimal model can be obtained by tuning hyperparameters, such as hidden layer size, activation function, optimizer, and class weight. The fitting model can be obtained by minimizing the error or loss function. We expected that the more optimal screening results could be obtained with these different methods than those with only one method. Each model had a different range of results. Thus, we could reduce the number of potential compound candidates by analyzing the overlap of prediction results from those three machine learning methods.

### Building the prediction model

The first step in building the model was normalization. First, the protein with dipeptide descriptor data was normalized using sklearn.preprocessing. MinMaxScaler function from the scikit-learn package in Python [35]. This scaler was then dumped to be used for the test protein data; the drug with PubChem fingerprint didn’t need to pass the normalization since the fingerprint was already in binary (0/1) format. The drug compounds and target data were then combined. We also added the class label according to the drug-target interaction data we got from SuperTarget. The training dataset consisted of 685 samples with interactions (positive class) and 39,180 samples with those unknown interactions (negative class). Thus, this dataset was actually unbalanced with the ratio between the positive and negative datasets of 1:57. Random oversampling with replacement was applied to the 685 positive datasets to obtain 10,578 samples of positive data.

Further, random under-sampling was applied to 39,180 negative datasets to reduce this dataset to 30% of the total negative dataset, to 11,754 samples of negative data. Thus, we had a total of 22,332 samples. These samplings were performed five times to obtain five random datasets, which consisted of 22,332 samples. Next, we randomly chose 70% of the total samples as a training dataset and 30% of them as a validation dataset. In the feature space, we had five matrixes of 15,632 × 1281 as the training set and 6,700 × 1281 as a validation set.

**Fig. 3:**
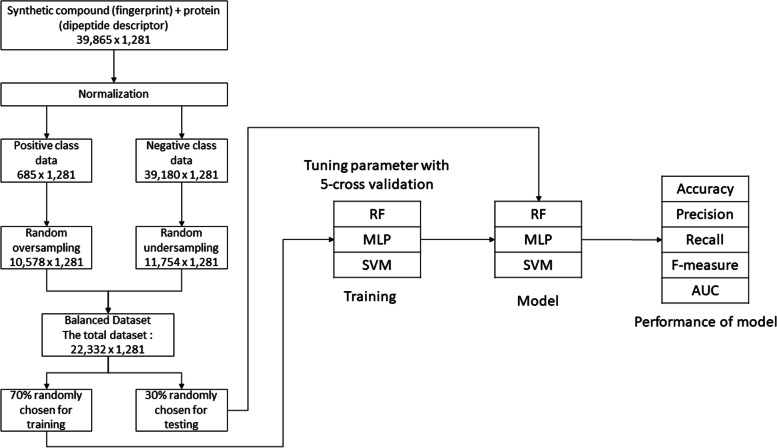
a schematic representation of model training and validation approaches for our proposed machine learning methodology

One of these five datasets was tuned for MLP, random forest (RF), and support vector machine (SVM) using a grid search technique with 5-fold cross-validation implemented using the grid search function from the scikit-learn package in Python. The grid search then saved the best parameters tuned based on the result of AUC from the cross-validation used inside the function. Four other models were built with the hyperparameter tuned from the first model. Next, the resulting models of each method were validated using the validation dataset. The performance results, including accuracy, precision, recall, f-measure, and area under curve (AUC), were calculated. Figure 3 shows the schema of our approach.

### Predicting Indonesian herbal compounds

**Fig. 4:**
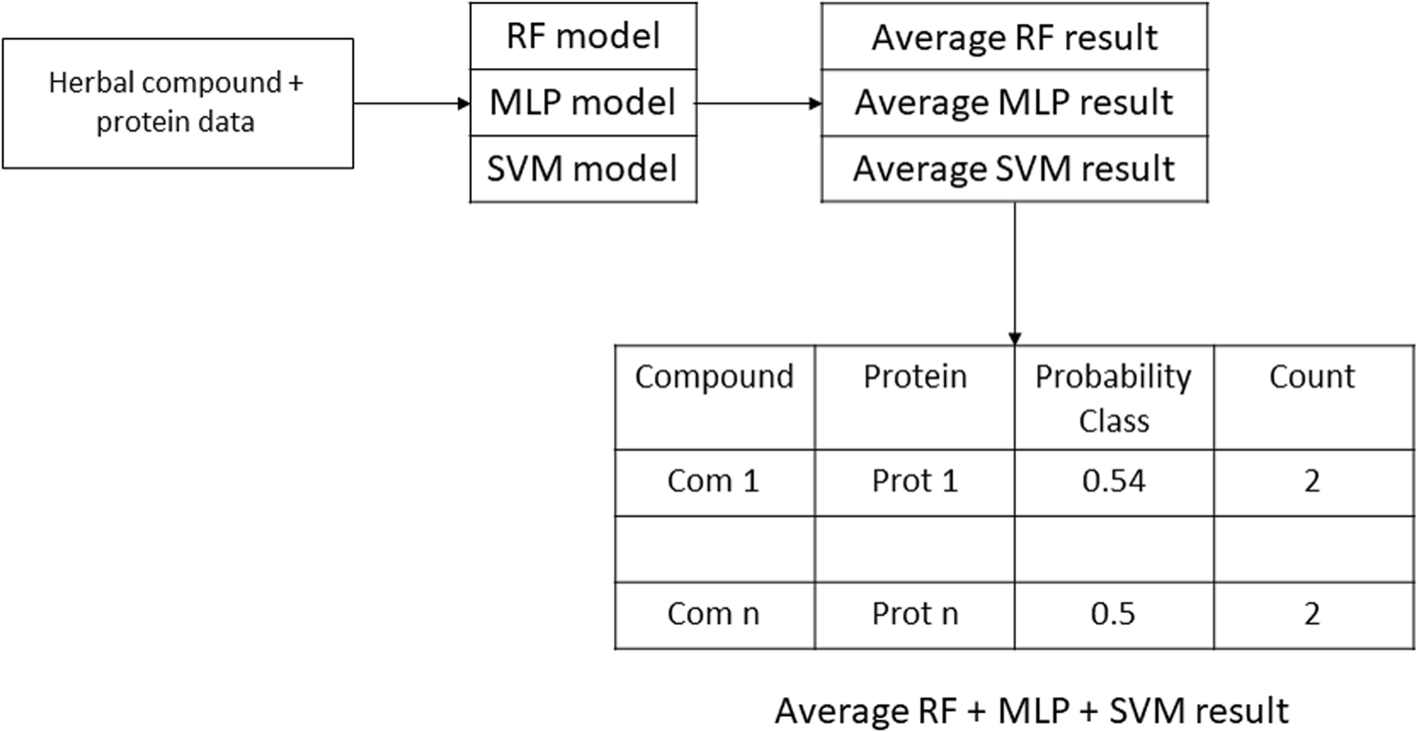
The scheme of predicting Indonesian herbal compounds using the optimal and validated model that was generated by RF, MLP, and SVM in training and validation phase. The class probability score was averaged from three methods. The counter was conducted to indicate the number of methods which predict positive results. The decision was determined based on the criteria of the class probability ≥ 0.5 or at least predicted by two methods.

The prediction of the Indonesian herbal compounds was conducted using five models of each method (MLP, RF, SVM). We used Indonesia herbal compounds collected from the HerbalDB database. These herbal compounds had no label. Thus, we predicted their interaction with the protein target using the validated model proposed in this study. For each method, the prediction result was obtained from the average probability score of the five prediction models. The herbal compounds that were predicted to have interactions with the protein target by at least two of the three methods or to have an average probability score ≥ 0.5 were used for subsequent analysis (Fig. 4).

### Pharmacophore modeling

Pharmacophore is defined as the interaction patterns of bioactive molecules with their target represented by a three-dimensional (3D) abstract feature arrangement that determines the types of interaction rather than specific functional groups. These types of interaction can, for example, include the formation of hydrogen bonds, charged interactions, metal interactions, or hydrophobic (H) and aromatic (AR) contacts [36]. Pharmacophore models can be generated using two different approaches depending on the input data used for model construction. In the structure-based approach, the interaction pattern of a molecule and its targets are directly extracted from the target ligand complex that is determined experimentally [37]. In the case of ligand-based modeling, the three-dimensional (3D) structures of two or more known active molecules are aligned, and common pharmacophore features shared among these training set molecules are identified. In the ligand-based approach, all the general chemical features of the pharmacophores should be considered essential, whereas the structure-based approach can consider whether the chemical features of a molecule are directly involved in ligand binding [38].

In our pharmacophore modeling approach, we used two methods—structure-based drug design (SBDD) and ligand-based drug design (LBDD)—using a one-month-free trial of LigandScout 4.3 software [38]. Based on a comparative analysis of eight pharmacophore tools, such as Catalyst, MOE, Pharmer, Unity, POT, LigandScout, Pharao, and Phase, we analyzed the compound library enrichment. The analysis of algorithm combinations showed that LigandScout was capable of improving the enrichment of other algorithms. In particular, LigandScout seemed to be complementary, as there was an improvement of both enrichment factors if it was used in a consecutive screening pipeline [39].

Pharmacophore modeling methods were conducted on macOS Mojave version 10.14.6; 2,3GHZ Intel Core i9 Processor, with a 16-GB 2400 MHz DDR4 memory. Parameter for conformations generations were set such as maximum number of conformations set as 100 and RMS threshold set as 0.7. For library clustering settings parameters were set as follow: similarity measure set as pharmacophore RDF-code similarity with maximum number of conformations 3 and cluster distance 0.4. For ligand-based pharmacophore creation, we used scoring function pharmacophore fit and atom overlap, pharmacophore type merged feature pharmacophore, number of omitted features were set as 4 and maximum number of pharmacophore model set as 10. Virtual screening mode for ligand-based pharmacophore was performed using scoring function pharmacophore-fit with screening mode match all query features, retrieval mode stop after first matching conformation and maximum number of omitted features was set to 0 from 6 pharmacophore features of the best pharmacophore model.

For structure-based methods, we used the 3D structure of SARS-CoV-2 main protease, which could be downloaded from Protein Data Bank (PDB) with ID code 6LU7 [40]. We chose the pharmacophore sites of the native ligand and identified the pharmacophore features. Next, LigandScout performed screening of medicinal plant compounds from HerbalDB based on the similarity of pharmacophore of the native ligand of SARS-CoV-2 main protease. Virtual screening mode for structure-based pharmacophore was performed using scoring function pharmacophore-fit with screening mode match all query features, retrieval mode stop after first matching conformation and maximum number of omitted features was set to 7 from 13 pharmacophore features of N3 native ligand.

Using the LBDD method, we collected 45 known SARS, MERS, and SARS-CoV-2 repurposing drug therapies from the literature and used them as datasets (Additional file 4). The molecules were downloaded from PubChem or prepared using MarvinSketch [41] and saved in .sdf format. The molecules were then separated into a training and test set, 15 molecules as a training set, and 30 molecules as a test set using the sklearn.model_selection.train_test_split method in Python and then inspected based on the pharmacophore features. For the pharmacophore modeling validation, we adapted the methods from Wolber and Langer (2015) and Seidel T (2017) [38, 42]. To validate the pharmacophore model results, we performed a validation process using decoy molecules that were generated using DUDE (www.dude.docking.org). We used DUDE to create decoy because DUD-E is one of the largest publicly available databases that offers the possibility to assess virtual screening programs’ efficiency in discriminating ligands from inactive compounds [43]. We prepared a library database in “Screening Perspective” window for active (test set) and decoy set molecules, and then saved the library in .ldb format. Furthermore, we screened active compounds (test set) and decoys based on each 10-model 3D pharmacophore. When the screening process was completed, a hit list of molecules that matched the pharmacophore was shown in the library view. Parameters of validation (ROC, AUC, and EF) were calculated to choose the best model [44–46]. Pharmacophore models that provided the best score of validation parameters were used for virtual screening against the Indonesian medicinal plant compounds database (HerbalDB).

### Molecular docking

Our molecular docking step resulted in compound candidates yielded by two approaches—machine learning and pharmacophore modeling—and used macromolecules of SARS-CoV-2 main protease (PDB ID: 6LU7). Molecular docking was conducted on macOS Mojave version 10.14.6; 2,3GHZ Intel Core i9 Processor, with a 16-GB 2400 MHz DDR4 memory. To validate the molecular docking, we performed the redocking process of the 6LU7 native ligand in AutoDock4 [47] software. The docking parameters used in this step were the Lamarckian genetic algorithm [48] with default docking parameter; binding site coordinates x = -9.732, y = 11.403, and z = 68.925; and grid box size 40 × 56 × 40. Autodock uses a Lamarckian genetic algorithm (LGA), which introduces a local search based on the traditional genetic algorithm, making it more efficient to determine the optimal docking [49]. With these parameters, we obtained the root-mean-square deviation (RMSD) value of the native ligand as < 2 Å [50] and then applied these parameters to other ligands. Docking results were carried out based on scoring and posing functions. Docking interactions were clustered to decide the Gibbs energy (ΔG), and optimum docking energy conformation and ligand-residue interaction were considered as the fine-docked pose.

## Results

### Machine learning

In the training phase, we conducted hyperparameter tuning for each method (RF, MLP, and SVM) to obtain the optimal prediction model. While the paper only focused on viral protein, the training data also used drug-target interaction in human protein to make a more representative and comprehensible model, thus the result we provide only explains the viral protein. The hyperparameter used for each method is provided as Additional file 5. The performance prediction model calculated using the validation dataset is shown in Table 3. The accuracies and f-measure value of the models were as high as 98% for all three methods. This indicates that our random over-sampling and random under-sampling performed well.

**Table 3:** The performance of each model calculated using 30% of dataset that was excluded from training set

Method	Performance Measure	Value
Multilayer Perceptron (MLP)	AUC	0.98405
	F-measure	0.98254
	Precision	0.96628
	Recall	0.99936
	Accuracy	0.98321
Random Forest (RF)	AUC	0.98734
	F-measure	0.98608
	Precision	0.97255
	Recall	1
	Accuracy	0.98665
Support Vector Machine (SVM)	AUC	0.99919
	F-measure	0.99911
	Precision	0.99847
	Recall	0.99975
	Accuracy	0.99915

Next, the models that had been optimized and validated were used to predict 400 Indonesian herbal compounds identified from HerbalDB. Table 4 shows some predicted results of herbal compounds that target 3CLPro, PLPro, and RdRp. The remaining prediction results can be found in Additional file 6. These candidate compounds have the potential to be compared to the pharmacophore modeling results. Some potential compounds resulting from a machine learning approach and pharmacophore modeling approach were further analyzed using molecular docking.

**Table 4:** The predicted potential compounds targeting 3CLPro, PLPro, and RdRp

No	Protein Target	Herbal Compound
1.	3CLPro	Amaranthine, 8-Methylthio-octyl glucosinolate, Arabinopyrano, Peonidin 3-(4’arabinosylglucoside), Quercetin 3-(2G-rhamnosylrutinoside), Sinigrin, Hesperidin, Myricetin-3-glucoside, (+)-2,3-Dihydro-9-hydroxy-2 [1-(6-sinapinoyl)beta-D-glucosyloxy-1-methylethyl]-7H-propanoat, Cyanidin-3-sophoroside-5-glucoside, Scutellarein-6,4’-dimethyl ether-7-(6”-acetylglucoside),, Spiraeoside, Glucoputranjivin, Isoforskolin, Kaempferol 3-alpha-D-arabinopyranoside
2.	PLPro	8-Methylthio-octyl glucosinolate, Sinigrin, Glucoputranjivin
3.	RdRp	8-Methylthio-octyl glucosinolate, Arabinopyrano, Peonidin 3-(4’arabinosylglucoside), Quercetin 3-(2G-rhamnosylrutinoside), Theviridoside, Sinigrin, Hesperidin, Myricetin-3-glucoside, , (+)-2,3-Dihydro-9-hydroxy-2 [1-(6-sinapinoyl)beta-D-glucosyloxy-1-methylethyl]-7H-propanoat y, Cyanidin-3-sophoroside-5-glucoside, Catalpol, Scandoside, Scutellarein-6,4’-dimethyl ether-7-(6”-acetylglucoside), Spiraeoside, Geniposide, Oleoside, Majoroside, Glucoputranjivin, Isoforskolin, Kaempferol 3-alpha-D-arabinopyranoside

### Pharmacophore modeling

#### Structure-based drug design (SBDD) methods

**Fig. 5:**
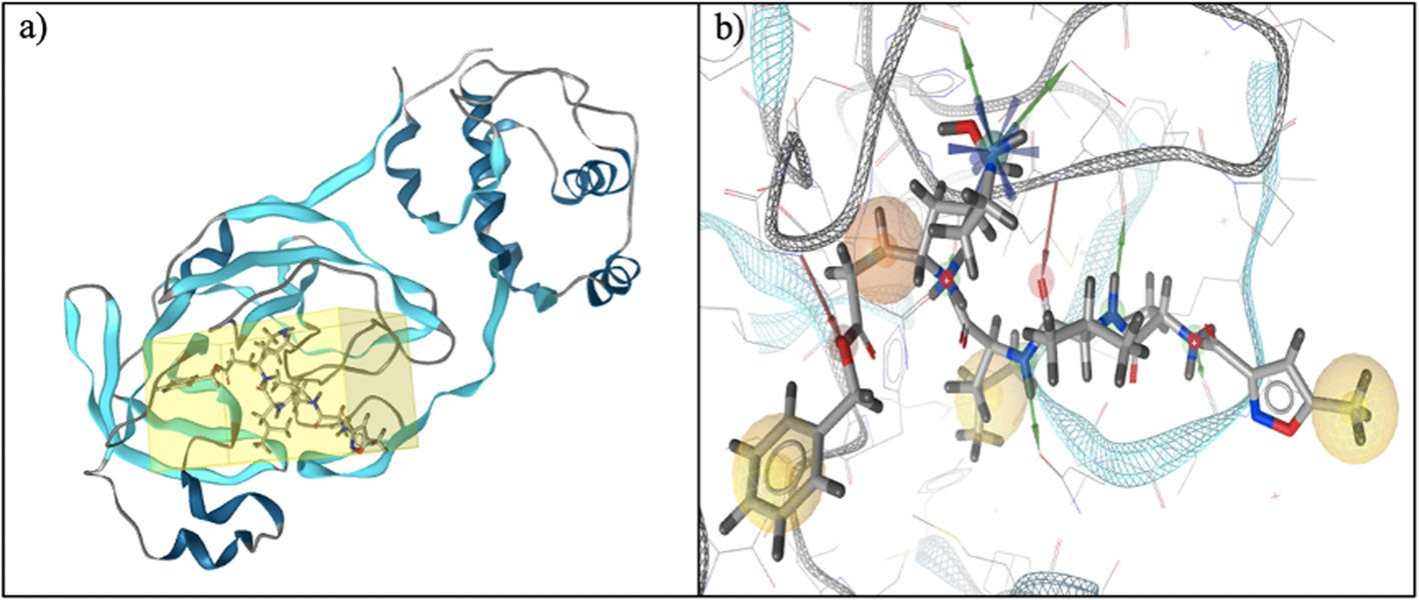
**a** 3D structure complex of the main protease and N3 inhibitor (N-[(5-Methylisoxazol-3-Yl)Carbonyl]Alanyl-L-Valyl-N~1~-((1R,2Z)-4-(Benzyloxy)-4-Oxo-1-{[(3R)-2-Oxopyrrolidin-3-Yl]Methyl}But-2-Enyl)-L-Leucinamide), **b** pharmacophore feature of the N3 inhibitor in the main protease

For SBDD methods, we analyzed 3CLpro (main protease) protein in its 3D structure (PDB ID: 6LU7) using LigandScout software. The complex of the main protease ligand and its pharmacophore features are shown in Fig. 5a and b, respectively. Based on that pharmacophore feature, we screened herbal compounds from the HerbalDB database. From this screening, we obtained eight hit compounds: kaempferol 3,4'-di-O-methyl ether (Ermanin); 4-Methylpentyl glucosinolate; 6-alpha-Hydroxyadoxoside; laurotetanine; orientanol E; 5-Methoxy-8-O-beta-D-glucosyloxypsoralen; rhamnetin 3-mannosyl-(1-2)-alloside; and 5,7,3',4'-tetrahydroxyflavanone 7-alpha-larabinofuranosyl-(1-6)-glucoside.

#### Ligand-based drug design (LBDD) methods

**Fig. 6:**
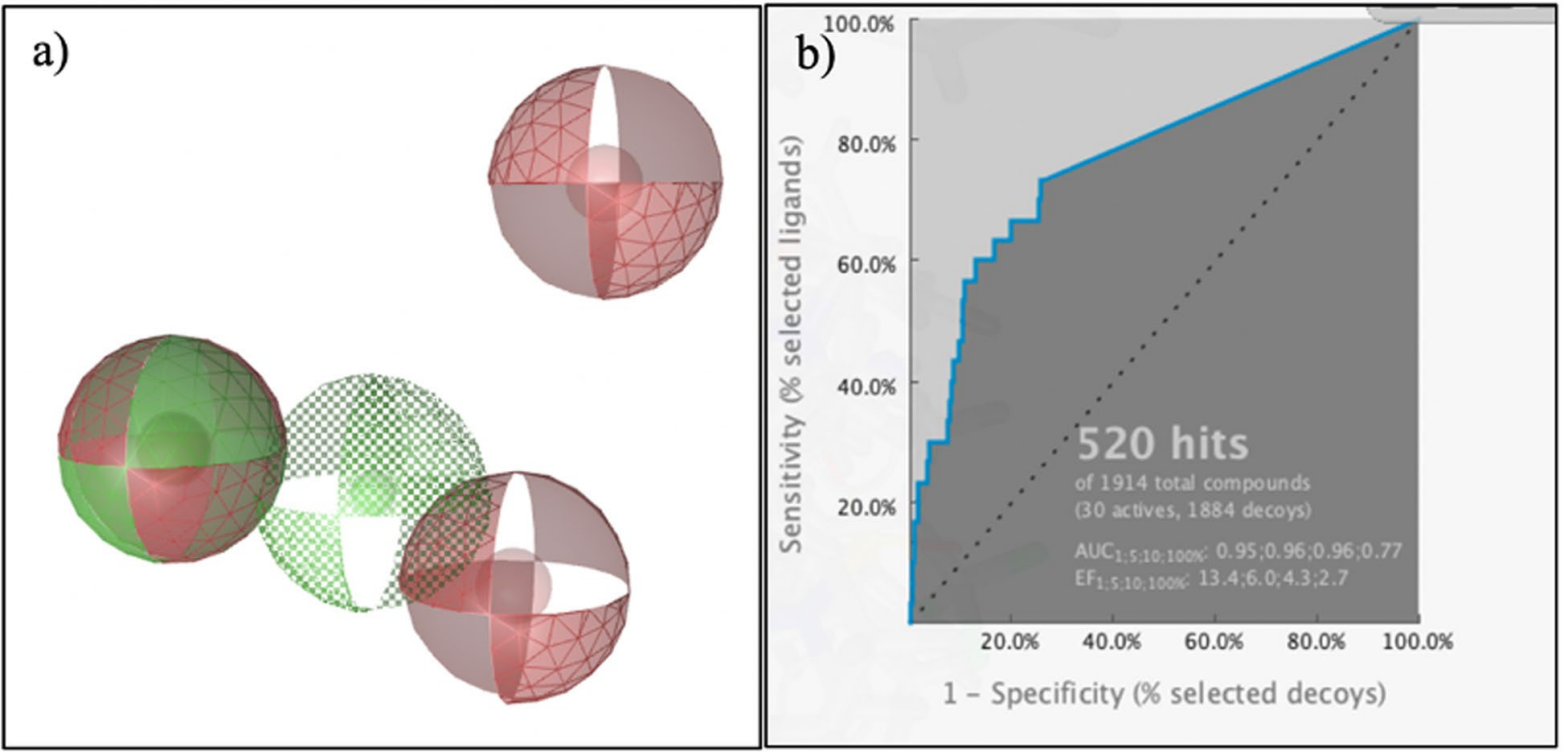
Pharmacophore model from LBDD analysis. **a** Pharmacophore feature of the best pharmacophore model, **b** validation parameters of the best pharmacophore model.

From LBDD analysis, we obtained ten pharmacophore models, and then we validated them to get the best pharmacophore model using decoy compounds. The validation parameters were AUC_100%_ and EF_1%_, the pharmacophore feature of the best pharmacophore model and its validation parameters are shown in Figure 6. The best pharmacophore model was model 4, with a hit rate of 27.17%, AUC_100%_ 0.77 and EF_1%_ 13.4 and it had five pharmacophore features consisting of three hydrogen bond acceptors (HBAs) and two hydrogen bond donors (HBDs). We screened the best pharmacophore model that was generated in the previous step against herbal compounds from HerbalDB and obtained the top 30 hit compounds. The top 30 hit compounds are shown in Table 5.

**Table 5:** The top-30 of hit compounds from LBDD methods

No	Compound Name	No	Compound Name
1	Kaempferol 3-alpha-D-arabinopyranoside	16	Catalpol
2	Isoforskolin	17	Cyanidin-3-sophoroside-5-glucoside
3	Glucoputranjivin	18	(+)-2,3-Dihydro-9-hydroxy-2 [1-(6-sinapinoyl)beta-D-glucosyloxy-1-methylethyl]-7H-propanoat
4	Loganic Acid	19	Myricetin 3-glucoside
5	Majoroside	20	Hesperidin
6	Oleoside	21	Azadirachtin A
7	Geniposide	22	1-Caffeoyl-beta-D-glucose
8	Glucobrassicin	23	Sinigrin
9	Spiraeoside	24	Theviridoside
10	Alizarin	25	Quercetin 3-(2G-rhamnosylrutinoside)
11	Morindone	26	Peonidin 3-(4’arabinosylglucoside)
12	Casuarinin	27	trans-p-Sinapoyl-b-D-glucopyranoside
13	Scutellarein-6,4’-dimethyl ether-7-(6”-acetylglucoside)	28	6,8-Di-C-beta-D-arabinopyranosyl apigenin
14	Scandoside methyl ester	29	8-Methylthio-octyl glucosinolate
15	beta-Glucogallin	30	Amaranthine

### Molecular docking

In the molecular docking step, we selected 3CLPro of SARS-COV-2 as a target because Mody et al [51] stated that the main protease is an excellent target since it is indispensable for viral replication. Before we started to dock the hit compounds to the 3CLpro protein, we redocked the native ligand to the 3CLpro binding site to confirm the suitability of the docking algorithm for virtual screening. The RMSD of re-docking of the 6LU7 native ligand was 0.34 Å, with respect to the co-crystallized ligand (Additional file 7). Although neither an effective antiviral drug nor a vaccine against COVID-19 is currently available, several reports have suggested that HIV-1 protease inhibitors, such as lopinavir, have the potential as SARS-CoV-2 protease inhibitors [6]. In an attempt to have reference values (positive control), we decided to consider lopinavir as a comparative standard for molecular docking. Based on machine learning, structure-based, and ligand-based pharmacophore results, we obtained 14 hit compounds that overlap from the machine learning and pharmacophore modeling approaches. Then, we used molecular docking to analyze the interaction between 3CLpro (main protease) protein in its 3D structure (PDB ID: 6LU7) with 14 hit compounds and used lopinavir as a positive control (Table 6).

**Table 6:** Molecular docking results of 14 hit (overlapped) compounds against the main protease of SARS-CoV-2

No	Compound name	Binding Energy (ΔG) (kcal/mol)	Sources
1	Cyanidin-3-sophoroside-5-glucoside	-6.52	*Brassica Oleracea* [52]*; Ipomoea Batatas* [53]*; Raphanus Sativus* [54]
2	Geniposide	-7.04	*Gardenia jasminoides* [55]
3	Hesperidin	-8.72	*Psidium guajava* [56] *Citrus aurantium* [56]
4	Isoforskolin	-6.88	*Coleus forskohlii* [57]
5	Kaempferol 3,4'-di-O-methylether (Ermanin)	-8.51	*Zingiber aromaticum* [58]
6	Majoroside	-7.03	*Plantago major* [59]
7	Myricetin-3-glucoside	-8.26	*Moringa oleifera* [60]
8	Oleoside	-6.52	*Oleaceae familia (e.g. Jasminum sambac)* [61]
9	Peonidine 3-(4’-arabinosylglucoside)	-8.52	*Ipomoea fistulosa* [62]
10	Quercetin 3-(2G-rhamnosylrutinoside)	-8.56	*Clitoria Ternatea* [63]
11	Rhamnetin 3-mannosyl-(1-2)-alloside	-8.48	*Moringa oleifera* [64] *Cassia alata* [65]
12	Sinigrin	-5.19	*Brassica nigra* [66]
13	Spiraeoside	-7.97	*Filipendula ulmaria* [67]
14	Theviridoside	-7.13	*Thevetia peruviana* [68]
15	Lopinavir	-9.41	Antiviral drug (positive control)

**Fig. 7:**
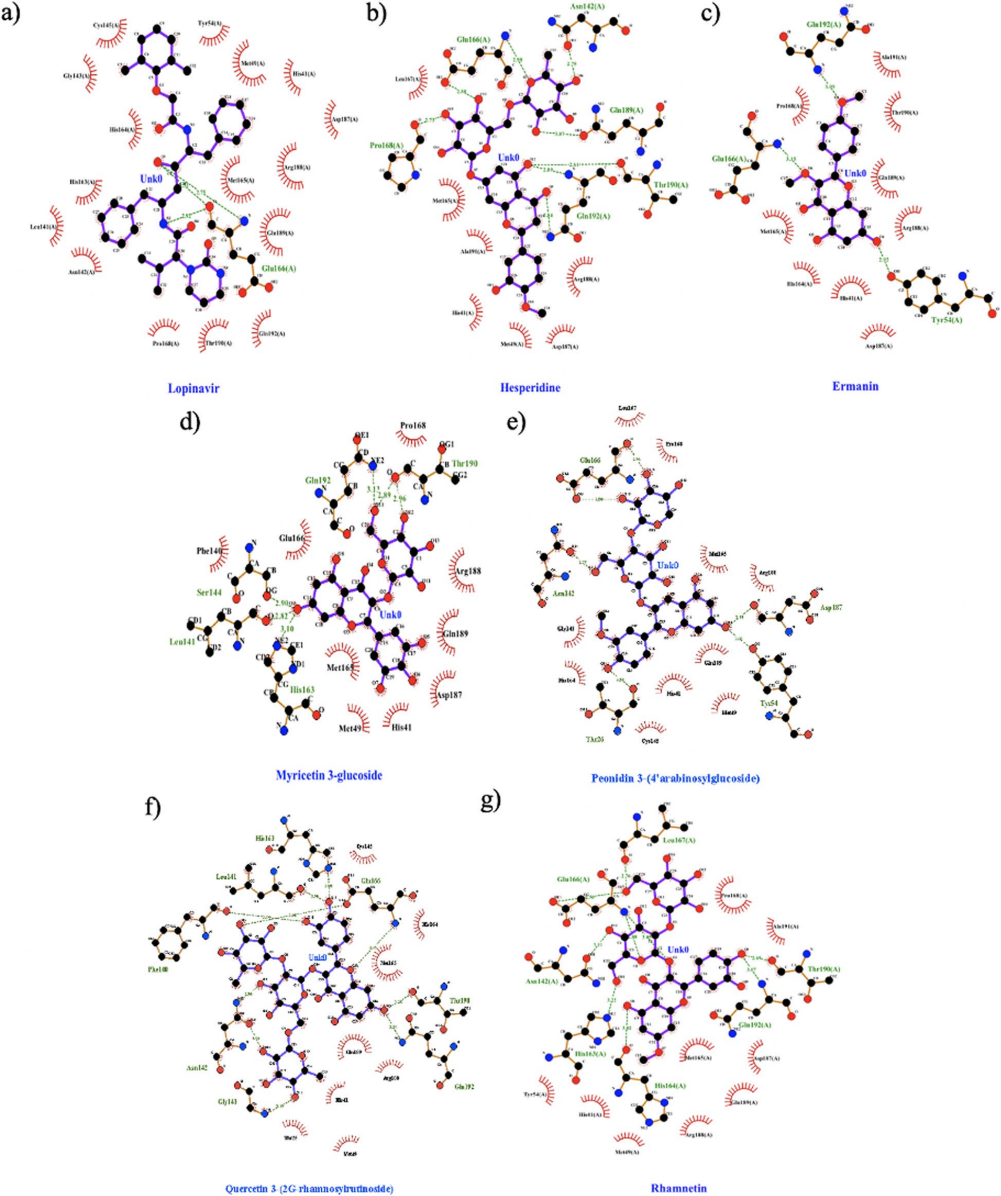
Interaction of ligands with receptor (3CLpro / main protease); red quarter circles were residue of protein that have non-covalent bond interaction with ligand; residues that written in green colour were residue which had hydrogen bonds interaction with ligand (written with its distance as well). **a** Lopinavir; **b** Hesperidin; **c** Kaempferol-3,4'-di-O-methyl ether (Ermanin); **d** Myricetin-3-glucoside; **e** Peonidine 3-(4’-arabinosylglucoside); **f** Quercetin 3-(2G-rhamnosylrutinoside); **g** Rhamnetin 3-mannosyl-(1-2)-alloside (visualization software using LigPlot [69])

From the molecular docking results, the tested compounds showed various binding energies (ΔG). Compounds that had binding energy close to that of lopinavir (positive control) were hesperidin, kaempferol-3,4'-di-O-methyl ether (Ermanin); myricetin-3-glucoside, peonidin 3-(4’-arabinosylglucoside); quercetin 3-(2G-rhamnosylrutinoside); and rhamnetin 3-mannosyl-(1-2)-alloside. Hesperidin showed the lowest binding energy (-8.72 kcal/mol) and was close to lopinavir’s binding energy (-9.41 kcal/mol). As shown in Fig. 7, lopinavir had a hydrogen bond with Glu166, which is an essential residue for maintaining the S1 pocket in the right shape and the enzyme in the active conformation [70]. Hesperidin, kaempferol-3,4'-di-O-methyl ether (Ermanin), quercetin 3-(2G-rhamnosylrutinoside), peonidin 3-(4’-arabinosylglucoside), and rhamnetin 3-mannosyl-(1-2)-alloside had hydrogen bonds with Glu166 residue as well. Lopinavir also had a binding interaction with the catalytic dyad (Cys-145 and His-41) of SARS-CoV-2, as well as six other compounds. The catalytic dyad is functionally essential residues (Cys-145 and His-41) that display stable behavior [71].

## Discussion

The SARS-CoV-2 virus is still emerging around the world. The number of infected people continues to grow, and there is still no definitive therapy that has been approved for effective treatment. Finding broad-spectrum inhibitors that could reduce the effects of coronavirus infection in humans remains a challenging research focus. Given the time-consuming nature of developing and registering antiviral drugs, drug repurposing is a shortcut to identifying a cure for the disease. Most approved drugs have sufficient activity and dosage, and their safety and ADME situation are well known.

Despite consistent effort in the research of conventional medicine, Indonesia, which has mega biodiversity, has potential herbal compounds that could be alternatives SARS-CoV-2 inhibitors. To obtain potential herbal compounds using a computational approach, we have to be careful about the research methods. We should not use our personal preferences of certain herbs, as it would lead to a subjective decision on the research results, particularly when the computational approach only uses the molecular docking method. Molecular docking is a powerful tool for pharmaceutical research that have been in development for decades, although there is a limitation of docking accuracy due to relatively simple scoring functions.

Additionally, entropic factors are generally not captured well by scoring based on a single structure. As a result, structure-based ligand screening by docking often generates a large number of false positive hits [72]. To minimize the false positive hits by conducting research with molecular docking only, we tried to use two different approaches in generating the prediction model before we performed the virtual screening on HerbalDB compounds. In this study, we used machine learning and pharmacophore modeling methods that are complementary to each other to generate a more accurate prediction model.

The machine learning approach was used to perform big data analysis using a DTI dataset curated from the literature and public domain database. This approach used pharmacological features obtained by integrating both the chemical space of compounds and the omics space of target proteins [73]. Cheminformatic studies found that machine learning approaches, such as similarity measures [74], bipartite graphs [75] and some classification techniques were useful in finding interactions between drugs and their protein targets [11, 76]. Most of the classification model was built for single-target protein drug problems. For instance, SVM, as one of the machine learning methods, can be employed to classify whether a compound is drug-like or non-drug-like [11]. Decision tree and neural network have also been attempted to distinguish drug-like compounds from non-drug-like compounds [77–79]. These approaches showed a maximum accuracy of up to 83% from a large dataset. In this study, the enhancement of those machine learning methods was done to classify whether a drug compound has a protein target or not.

Dealing with the issue of high dimensional data in the feature space formed by the fingerprint of compounds and the dipeptide descriptors of proteins, many papers show the effectiveness of the embedded capacity of several classifiers [80], such as SVM [81], neural network-based algorithm (MLP) [82], and decision tree-based algorithm (RF) [83] to discard input features. Embedded methods have the advantage that they include interaction with the classification model [78]. In the random forest method, we tuned *mtry* that indicated a random selection of *m* predictors as a subset of split candidates from the full set of predictors when building trees. Thus, in RF, the high dimensionality is reduced by choosing *mtry* smaller than the number of features. However, for MLP and SVM, even though both of them were able to handle non-linearity (SVM with the kernel; MLP with multilayers), they are still vulnerable to spurious correlation. This meant there were some features that appeared to be highly correlated in training data, but less sensitive in real prediction using testing data. The prediction model generated by SVM showed the tendency. Although all models generated by the three methods (MLP, RF, and SVM) had high accuracy in the validation step, the SVM failed to predict herbal compounds. Only very few herbal compounds could be predicted by SVM compared to MLP and RF.

The unbalanced dataset probably also contributed to the performance of SVM. Random oversampling was not adequate to improve the performance of SVM because the number of different support vectors did not increase. Thus, the hyperplane was not improved. Oversampling with replacement did not affect the distribution of the support vector but affected class probability. Therefore, in this case, RF is more robust than other methods because the oversampling increased the class probability that was required for splitting when building the tree.

Our criteria to choose the high probability drug-target interaction demanded that the herbal candidate compounds’ should be predicted by at least two methods or should have an average probability score ≥ 0.5. Then, the predicted results would be filtered again by comparing them to ligand-based and structure-based pharmacophore methods. We included two machine learning methods (i.e., MLP and RF) in the acceptance criteria. The SVM model was excluded because it was not highly prodective in comparison with the selected methods. Biswas et al [84] stated, the machine learning approach can be used to predict DTI with insufficient known ligands. Thus, our approach provided layered filtering to conduct more objective and optimal virtual screening.

Based on structure-based pharmacophore modeling of N3 inhibitor in 3CLPro binding site, 13-feature pharmacophores were identified such as 4 hydrophobic features, 2 hydrogen bond acceptor features, 6 hydrogen bond donor features and 1 positive ionizable area feature. Some amino acid residues such as Met49, Ala191, Thr25 and Thr26 have been identified as hydrophobic features. Five amino acid residues including Thr190, Gln189, Glu166, His164, and Phe140 play a role in hydrogen bond donor interactions with N3 inhibitor. Interestingly, Glu166 has a hydrogen bond acceptor, hydrogen bond donor and positive ionizable interactions with the -NH functional group of N3 inhibitor. The other amino acids like Gly143 have been identified as hydrogen bond acceptor pharmacophore features. A pharmacophore is the pattern of features of a molecule that is responsible for a biological effect, which captures the essential notion that a pharmacophore is built from features rather than defined chemical groups. Every type of atom or group in a molecule that exhibits specific properties related to molecular recognition can be reduced to a pharmacophore feature. These molecular patterns can be labeled as hydrogen bond donors or acceptors, cationic, anionic, aromatic, or hydrophobic, and any possible combinations. Pharmacophore models are very suitable as queries for virtual screening of databases. Pharmacophore models are often utilized as a filter to identify compounds that fulfill the simple geometric and chemical functionality requirements of the query, before more complicated and computationally demanding approaches such as molecular docking [85]. Thus, using two approaches in the methodology—machine learning and pharmacophore modeling—increased our confidence level of the predicted candidate compounds. In this case, the best pharmacophore model from ligand-based is obtained with Enrichment Factor 1% (EF1%) value 13.4 and AUC100% value 0.77 from total 30 actives and 1884 decoys.

Based on Jin et al (2020) study, N3 molecule exhibits a very potent inhibition of SARS-CoV-2 Mpro, such that the measurement of Ki and k3 was not feasible. When very rapid inactivation occurs, kobs/[I] was used to evaluate the inhibition as an approximation of the pseudo-second-order rate constant (k3/Ki). They determined the value of kobs/[I] of N3 for SARS-CoV-2 Mpro as 11,300 ± 880 M−1 s−1, which suggests that this acceptor was markedly inhibited N3 displayed inhibition against SARS-CoV-2 with individual half-maximal effective concentration (EC50) values of 16.77 μM [40].

Our layered virtual screening of HerbalDB obtained 14 compounds that had overlapping results from the two methods. Molecular docking algorithms are often calibrated against experimental ligand-protein complex training sets, and the accuracy of these docking programs is often highly dependent on the training sets used [84]. In this case, it is essential to ensure that the docking software used for virtual screening can replicate the binding mode of a known experimental inhibitor for the enzymes studied. From molecular docking analysis, we obtained six potential compounds: hesperidin, kaempferol-3,4'-di-O-methyl ether (Ermanin); myricetin-3-glucoside; peonidin 3-(4’-arabinosylglucoside); quercetin 3-(2G-rhamnosylrutinoside); and rhamnetin 3-mannosyl-(1-2)-alloside, which were predicted as inhibitors of the 3CLpro protein of SARS-CoV-2.

From the 14 compounds we found before, myricetin-3-glucoside and rhamnetin 3-mannosyl-(1-2)-alloside were found in *Moringa oleifera* (Table 6). Moringa oleifera Lam. is one of the vastly used plant whose various parts (leaf, fruit, seeds etc.) are included in regular diet for their multiple ability of combating several health issues. Several studies were reported regarding the antiviral activity of *M. oleifera* plant, a pronounced bioprospective aspirant. The plant is known to be used in many traditional medicines and pharmacopeias against an array of medical conditions that include malaria, diabetes, skin infection, tuberculosis, anemia, headaches, epilepsy, sexually transmitted diseases and so on. In African traditional medicine, the plant is popularly used against AIDS and related secondary infections associated with HIV. It showed significant activities against viruses like HIV, HSV, HBV, EBV, FMDV and NDV [86].

The usage of medicinal plants as a key component of complementary and alternative medicine, has acquired renewed interest in developed countries. Medicinal crops are designated as cultivated / semi cultivated plants for prevention/ treatment of human diseases. Large numbers of medicinal plants, which have long history of being utilized in tropical and subtropical regions of the world. They have a long history of being utilized as medicines to control human diseases [87]. One of the medicinal crops in Indonesia is guava (*Psidium guajava*), which can be harvested continuously during the year. In Indonesia, production of guava in the year 2018 is 230,697 tons, with a growth rate from the year 2017 to 2018 of 15.06% [88]. Guava is consumed not only as food but also as a medicinal plant in subtropical areas around the world due to its pharmacologic activities. Based on the *Herbal Regulation as Healthy Supplement for Fighting COVID-19* in Indonesia published by the Indonesian Food and Drug Authority (BPOM) (May 2020), we can consume 1–4 fruits guava fruits per day (55-100 gram/fruit) which contain vitamin C 228.3 mg in 100 gram fruit. For the administration, guava can be eaten directly or processed as juice. There is no toxicity for long-term consumption of guava; overall, this herbal is safe to use as a daily nutritional supplement [89]. Phenolic compounds from guava has been proved to be immunomodulators and antioxidants [90].

Guava is well known to have several flavonoid compounds, such as myricetin, quercetin, luteolin, kaempferol, isorhamnetin [91], and hesperidin [92]. These compounds were also identified in our study, although without the aglycones. Luteolin is known as a furin protein inhibitor [93], which is predicted to be one of the enzymes that break down Coronavirus S (spike) protein as in MERS into units S1 and S2 [94]. In the S1 unit, there is a receptor binding domain (RBD) where the ACE2 peptidase binds so that the virus can bind to the host [94]. Hesperidin/hesperitin compounds in the *in silico* study are known to inhibit RBD domain binding of the SARS-CoV-2 Spike protein with ACE2 receptors in humans so that it is predicted to inhibit the entry of the SARS-CoV-2 potentially [5]. It is also known that luteolin is a neuraminidase inhibitor as well as oseltamivir, which is currently one of the drugs used in the CDC protocol for COVID-19 standard treatment. Hesperitin (the form of hesperidin aglycone) and quercetin are known to also act as inhibitors of 3CLpro [95, 96]. Other compounds in guava, such as myricetin, are known to act as SARS coronavirus helicase inhibitors [97]. Kaempferol has the potential to be a non-competitive inhibitor of 3CLPro and PLpro as well as quercetin [98]. Another interesting fact is that kaempferol acts as a modulator of autophagy, which can be utilized in strategies to inhibit the SARS-CoV-2 virus.

## Conclusions

We used layered virtual screening with machine learning and pharmacophore modeling approaches to determine the potential candidate compounds in Indonesian herbal medicine as a COVID-19 supportive therapy. Our methods provide an objective and optimal virtual screening outcome, and avoids subjective decision making on research results. The accuracies and f-measure values of the machine learning models are as high as 98%. The best pharmacophore model achieved a hit rate of 27.17%, AUC100% of 0.77, and EF1% of 13.4. This approach led to prioritization of potential anti-SARS-CoV-2 herbal compounds including hesperidin, kaempferol-3,4'-di-O-methyl ether (Ermanin); myricetin-3-glucoside, peonidin 3-(4’-arabinosylglucoside); quercetin 3-(2G-rhamnosylrutinoside); and rhamnetin 3-mannosyl-(1-2)-alloside. All of these compounds docked well in 3D structure of the main protease of SARS-CoV-2, known as 3CLPro with binding energies comparable to that of lopinavir's (positive control). Thus, *Moringa oleifera* and *Psidium guajava* that consist of those compounds, could be used as COVID-19 herbal preventive or palliative treatments. Additionally, our approach could be successfully applied for other drug discovery projects and is expected to speed up the screening process for adjuvant herbal therapies.

## Supplementary Information

**Additional file 1.** List of Potential Virus-based Drug Related to COVID-19.

**Additional file 2.** List of Potential Human-based Drug Related to COVID-19.

**Additional file 3.** Training dataset of Drug Target Interactions.

**Additional file 4.** Training and test dataset for ligand-based method.

**Additional file 5.** The optimal hyper-parameter values of each model.

**Additional file 6.** The prediction results of herbal compounds.

**Additional file 7.** Redocking result for 6LU7 native ligand.

## Data Availability

All data and source codes of the machine learning approach used in this research can be accessed at https://github.com/TropBRC-BioinfoLab/virtual-screening-covid19. HerbalDB datasets are available on request to the authors.

## Abbreviations

3CLPro: 3C-like protease
AUC: Area Under the Curves
COVID-19: Coronavirus Disease 2019
DTI: Drug Target Interactions
DUDE: Database of Useful (Docking) Decoys
EF: Enrichment Factor
LBDD: Ligand-based Drug Design
MLP: Multilayer Perceptron
MERS-CoV: Middle East Respiratory Syndrome coronavirus
PDB: Protein Data Bank
PLPro: Papain-like Protease
RdRp: RNA-dependent RNA Polymerase
RF: Random Forest
SARS-CoV: Severe Acute Respiratory Syndrome coronavirus
SARS-CoV-2: Severe Acute Respiratory Syndrome coronavirus 2
SBDD: Structure-based Drug Design
SVM: Support Vector Machine
