# Supplementary material for: Virtual screening of Indonesian herbal compounds as COVID-19 supportive therapy: machine learning and pharmacophore modeling approaches
Source: BMC Complement Med Ther. 2022 Aug 3;22:207. doi: 10.1186/s12906-022-03686-y (PMC9347098; doi:10.1186/s12906-022-03686-y)
Supplement: Supplementary file 1 — Additional file 1. List of Potential Virus-based Drug Related to COVID-19. [file 12906_2022_3686_MOESM1_ESM.docx]

**Additional File 1** List of Potential Virus-based Drug Related to COVID-19.

| **Drug name** | **Reference** | **Drug name** | **Reference** |
| --- | --- | --- | --- |
| Alfuzosin | [1] | Idarubicin | [1] |
| Almitrine | [1] | Indinavir | [1] |
| Amodiaquine | [2] | Iopromide | [1] |
| Amprenavir | [1] | Isotretinoin | [1] |
| Atazanavir | [1] | Itraconazole | [1] |
| Atovaquone | [1] | Lavodropropizine | [1] |
| Benzylpenicilloyl G | [1] | Loperamide | [3] |
| Bromocriptine | [1] | Lopinavir | [3]–[7] |
| b-thymidine | [1] | Lutein | [1] |
| Candoxatril | [1] | Lymecycline | [1] |
| Carvedilol | [1] | Masoprocol | [1] |
| Cefpiramide | [1] | Mefloquine | [15] |
| Ceftibuten | [5] | Mimosine | [5] |
| Cefuroxime | [5] | Montelukast | [5] |
| Chenodeoxycholic acid | [5] | Nafamostat | [30,31] |
| Chloramphenicol | [5] | Nelvinafir | [5] |
| Chlorhexidine | [5] | Nepafenac | [5] |
| Cilastatin | [5] | Nicardipine | [5] |
| Cobicistat | [6] | Novobiocin | [5] |
| Conivaptan | [5] | Oseltamivir | [6] |
| Cortisone | [5] | Oxytetracycline | [5] |
| Cromolyn | [5] | Pancuronium bromide | [5] |
| Dabigatran etexilate | [5] | Penciclovir | [30] |
| Dantrolene | [5] | Phenethicillin | [5] |
| Darunavir | [6] | Pioglitazone | [5] |
| Demeclocycline | [5] | Progabide | [5] |
| Diphenoxylate | [5] | Remdesivir | [35,30,32–34] |
| Disulfiram | [35] | reproterol | [5] |
| Doxycycline | [5] | Resveratrol | [36] |
| Estradiol valerate | [5] | Ribavirin | [4,5,30,37–40] |
| Famotidine | [5] | Ritonavir | [25,26,35,41] |
| Favipiravir | [23,25] | Saquinavir | [5] |
| Fenoterol | [5] | Silybin | [5] |
| Flavin mononucleotide | [5] | Sulfasalazine | [5] |
| Floxuridine | [5] | Telmisartan | [5] |
| Fludarabine | [5] | Tibolone | [5] |
| fosamprenavir | [5] | Tigecycline | [5] |
| Galidesivir | [26] | Tipranavir | [5] |
| Gemcitabine Hydrochloride | [29] | Valganciclovir | [5] |

**References**

[1] C. Wu *et al.*, “Analysis of therapeutic targets for SARS-CoV-2 and discovery of potential drugs by computational methods,” *Acta Pharm. Sin. B*, no. PG-, 2020, doi: https://doi.org/10.1016/j.apsb.2020.02.008.

[2] P. B. Madrid *et al.*, “A Systematic Screen of FDA-Approved Drugs for Inhibitors of Biological Threat Agents,” *PLoS One*, vol. 8, no. 4, 2013, doi: 10.1371/journal.pone.0060579.

[3] A. H. De Wilde *et al.*, “Screening of an FDA-approved compound library identifies four small-molecule inhibitors of Middle East respiratory syndrome coronavirus replication in cell culture,” *Antimicrob. Agents Chemother.*, vol. 58, no. 8, pp. 4875–84, 2014, doi: 10.1128/AAC.03011-14.

[4] T. P. Sheahan *et al.*, “Comparative therapeutic efficacy of remdesivir and combination lopinavir, ritonavir, and interferon beta against MERS-CoV,” *Nat. Commun.*, vol. 11, no. 1, p. 222, 2020, doi: 10.1038/s41467-019-13940-6.

[5] T. K. Warren *et al.*, “Protection against filovirus diseases by a novel broad-spectrum nucleoside analogue BCX4430,” *Nature*, vol. 508, no. 7496, pp. 402–5, 2014, doi: 10.1038/nature13027.

[6] J. F. W. Chan *et al.*, “Treatment with lopinavir/ritonavir or interferon-β1b improves outcome of MERSCoV infection in a nonhuman primate model of common marmoset,” *J. Infect. Dis.*, vol. 212, no. 12, pp. 1904–1913, 2015, doi: 10.1093/infdis/jiv392.

[7] U. J. Kim, E. J. Won, S. J. Kee, S. I. Jung, and H. C. Jang, “Combination therapy with lopinavir/ritonavir, ribavirin and interferon-a for Middle East respiratory syndrome,” *Antivir. Ther.*, vol. 21, no. 5, pp. 455–459, 2016, doi: 10.3851/IMP3002.

[8] S. C. Lin, C. T. Ho, W. H. Chuo, S. Li, T. T. Wang, and C. C. Lin, “Effective inhibition of MERS-CoV infection by resveratrol,” *BMC Infect. Dis.*, vol. 17, no. 1, p. 144, 2017, doi: 10.1186/s12879-017-2253-8.

[9] M. Wang *et al.*, “Remdesivir and chloroquine effectively inhibit the recently emerged novel coronavirus (2019-nCoV) in vitro,” *Cell Res.*, vol. 30, pp. 269–271, 2020, doi: 10.1038/s41422-020-0282-0.

[10] K. Ito *et al.*, “Geographic distribution and characteristics of genotype A hepatitis B virus infection in acute and chronic hepatitis B patients in Japan,” *J. Gastroenterol. Hepatol.*, vol. 31, no. 1, pp. 180–9, 2016, doi: 10.1111/jgh.13030.

[11] G. Li and E. De Clercq, “Therapeutic options for the 2019 novel coronavirus (2019-nCoV),” *Nat. Rev. Drug Discov.*, vol. 19, pp. 149–150, 2020, doi: 10.1038/d41573-020-00016-0.

[12] M. L. Agostini *et al.*, “Coronavirus susceptibility to the antiviral remdesivir (GS-5734) is mediated by the viral polymerase and the proofreading exoribonuclease,” *MBio*, vol. 9, no. 2, 2018, doi: 10.1128/mBio.00221-18.

[13] T. P. Sheahan *et al.*, “Broad-spectrum antiviral GS-5734 inhibits both epidemic and zoonotic coronaviruses,” *Sci. Transl. Med.*, vol. 9, no. 396, 2017, doi: 10.1126/scitranslmed.aal3653.

[14] A. J. Brown *et al.*, “Broad spectrum antiviral remdesivir inhibits human endemic and zoonotic deltacoronaviruses with a highly divergent RNA dependent RNA polymerase,” *Antiviral Res.*, vol. 169, 2019, doi: 10.1016/j.antiviral.2019.104541.

[15] M. H. Lin *et al.*, “Disulfiram can inhibit MERS and SARS coronavirus papain-like proteases via different modes,” *Antiviral Res.*, vol. 150, pp. 155–163, 2018, doi: 10.1016/j.antiviral.2017.12.015.

[16] D. Falzarano *et al.*, “Treatment with interferon-α2b and ribavirin improves outcome in MERS-CoV-infected rhesus macaques,” *Nat. Med.*, vol. 19, no. 10, 2013, doi: 10.1038/nm.3362.

[17] S. Shalhoub *et al.*, “IFN-α2a or IFN-β1a in combination with ribavirin to treat Middle East respiratory syndrome coronavirus pneumonia: A retrospective study,” *J. Antimicrob. Chemother.*, vol. 70, no. 7, 2015, doi: 10.1093/jac/dkv085.

[18] J. A. Al-Tawfiq, H. Momattin, J. Dib, and Z. A. Memish, “Ribavirin and interferon therapy in patients infected with the Middle East respiratory syndrome coronavirus: An observational study,” *Int. J. Infect. Dis.*, vol. 20, pp. 42–6, 2014, doi: 10.1016/j.ijid.2013.12.003.

[19] J. F. W. Chan *et al.*, “Broad-spectrum antivirals for the emerging Middle East respiratory syndrome coronavirus,” *J. Infect.*, vol. 67, no. 6, pp. 606–16, 2013, doi: 10.1016/j.jinf.2013.09.029.

[20] A. S. Omrani *et al.*, “Ribavirin and interferon alfa-2a for severe Middle East respiratory syndrome coronavirus infection: A retrospective cohort study,” *Lancet Infect. Dis.*, vol. 14, no. 11, pp. 1090–1095, 2014, doi: 10.1016/S1473-3099(14)70920-X.

[21] Y. M. Arabi *et al.*, “Treatment of Middle East Respiratory Syndrome with a combination of lopinavir-ritonavir and interferon-β1b (MIRACLE trial): Study protocol for a randomized controlled trial,” *Trials*, vol. 19, no. 1, 2018, doi: 10.1186/s13063-017-2427-0.
