# Supplementary material for: Virtual screening of Indonesian herbal compounds as COVID-19 supportive therapy: machine learning and pharmacophore modeling approaches
Source: BMC Complement Med Ther. 2022 Aug 3;22:207. doi: 10.1186/s12906-022-03686-y (PMC9347098; doi:10.1186/s12906-022-03686-y)
Supplement: Supplementary file 2 — Additional file 2. List of Potential Human-based Drug Related to COVID-19. [file 12906_2022_3686_MOESM2_ESM.docx]

**Additional File 2** List of Potential Human-based Drug Related to COVID-19

| **Drug name** | **Reference** |
| --- | --- |
| Chloroquine | [1–4] |
| Cyclosporine A | [5–7] |
| Imatinib mesylate | [2,8] |
| Dasatinib | [9] |
| Selumetinib | [10] |
| Trametinib | [10] |
| Rapamycin | [10] |
| Saracatinib | [11] |
| Promethazine | [1,2] |
| Thiethylperazine | [1,2] |
| Fluphenazine | [1,2] |
| Triflupromazine | [1,2] |
| Chlorpromazine | [1,2] |
| Mycophenolic acid | [12,13] |
| Silvestrol | [14] |
| LMT-28 | [15] |

**References**

1. Madrid PB, Chopra S, Manger ID, Gilfillan L, Keepers TR, Shurtleff AC, et al. A Systematic Screen of FDA-Approved Drugs for Inhibitors of Biological Threat Agents. PLoS One. 2013;8(4).

2. Kim UJ, Won EJ, Kee SJ, Jung SI, Jang HC. Combination therapy with lopinavir/ritonavir, ribavirin and interferon-a for Middle East respiratory syndrome. Antivir Ther. 2016;21(5):455–9.

3. Lin SC, Ho CT, Chuo WH, Li S, Wang TT, Lin CC. Effective inhibition of MERS-CoV infection by resveratrol. BMC Infect Dis. 2017;17(1):144.

4. Arabi YM, Alothman A, Balkhy HH, Al-Dawood A, AlJohani S, Al Harbi S, et al. Treatment of Middle East Respiratory Syndrome with a combination of lopinavir-ritonavir and interferon-β1b (MIRACLE trial): Study protocol for a randomized controlled trial. Trials. 2018;19(1).

5. Barnard DL, Day CW, Bailey K, Heiner M, Montgomery R, Lauridsen L, et al. Evaluation of immunomodulators, interferons and known in vitro SARS-CoV inhibitors for inhibition of SARS-CoV replication in BALB/c mice. Antivir Chem Chemother. 2006;17(5):275–84.

6. Pfefferle S, Schöpf J, Kögl M, Friedel CC, Müller MA, Carbajo-Lozoya J, et al. The SARS-Coronavirus-host interactome: Identification of cyclophilins as target for pan-Coronavirus inhibitors. PLoS Pathog. 2011;7(11).

7. Tanaka Y, Sato Y, Sasaki T. Suppression of coronavirus replication by cyclophilin inhibitors. Viruses. 2013;5(5):1250–60.

8. de Wilde AH, Raj VS, Oudshoorn D, Bestebroer TM, van Nieuwkoop S, Limpens RWAL, et al. MERS-coronavirus replication induces severe in vitro cytopathology and is strongly inhibited by cyclosporin A or interferon-α treatment. J Gen Virol. 2013;94(8):1749–60.

9. Sheahan TP, Sims AC, Leist SR, Schäfer A, Won J, Brown AJ, et al. Comparative therapeutic efficacy of remdesivir and combination lopinavir, ritonavir, and interferon beta against MERS-CoV. Nat Commun. 2020;11(1):222.

10. Coleman CM, Sisk JM, Mingo RM, Nelson EA, White JM, Frieman MB. Abelson Kinase Inhibitors Are Potent Inhibitors of Severe Acute Respiratory Syndrome Coronavirus and Middle East Respiratory Syndrome Coronavirus Fusion. J Virol. 2016;90(19):8924–33.

11. Kindrachuk J, Ork B, Hart BJ, Mazur S, Holbrook MR, Frieman MB, et al. Antiviral potential of ERK/MAPK and PI3K/AKT/mTOR signaling modulation for Middle East respiratory syndrome coronavirus infection as identified by temporal kinome analysis. Antimicrob Agents Chemother. 2015;59(2):1088–99.

12. Cheng KW, Cheng SC, Chen WY, Lin MH, Chuang SJ, Cheng IH, et al. Thiopurine analogs and mycophenolic acid synergistically inhibit the papain-like protease of Middle East respiratory syndrome coronavirus. Antiviral Res. 2015;115:9–16.

13. Shin JS, Jung E, Kim M, Baric RS, Go YY. Saracatinib inhibits middle east respiratory syndrome-coronavirus replication in vitro. Viruses. 2018;10(6).

14. Ahmed-Belkacem A, Colliandre L, Ahnou N, Nevers Q, Gelin M, Bessin Y, et al. Fragment-based discovery of a new family of non-peptidic small-molecule cyclophilin inhibitors with potent antiviral activities. Nat Commun. 2016;7.

15. Müller C, Schulte FW, Lange-Grünweller K, Obermann W, Madhugiri R, Pleschka S, et al. Broad-spectrum antiviral activity of the eIF4A inhibitor silvestrol against corona- and picornaviruses. Antiviral Res. 2018;150:123–9.
