# Supplementary material for: Virtual screening of Indonesian herbal compounds as COVID-19 supportive therapy: machine learning and pharmacophore modeling approaches
Source: BMC Complement Med Ther. 2022 Aug 3;22:207. doi: 10.1186/s12906-022-03686-y (PMC9347098; doi:10.1186/s12906-022-03686-y)
Supplement: Supplementary file 3 — Additional file 3. Training dataset of Drug Target Interactions. [file 12906_2022_3686_MOESM3_ESM.docx]

**Additional file 3.** Training Dataset of Drug Target Interactions

| **Compounds** | **Protein** |
| --- | --- |
| Arsentrioxide | AKT1_HUMAN |
| Arthrocine | AKT1_HUMAN |
| Celecoxib | AKT1_HUMAN |
| Dasatinib | AKT1_HUMAN; AAPK1_HUMAN; AAPK2_HUMAN; ABL1_HUMAN; ABL2_HUMAN; ACK1_HUMAN; ACV1B_HUMAN; ACVL1_HUMAN; ACVR1_HUMAN; BTK_HUMAN; CDK2_HUMAN; CDK5_HUMAN; CDK7_HUMAN; CDK8_HUMAN; CDK9_HUMAN; CLK1_HUMAN; CSF1R_HUMAN; CSK21_HUMAN; CSK_HUMAN; DAPK1_HUMAN; DDR1_HUMAN; EGFR_HUMAN; EPHA2_HUMAN; EPHB1_HUMAN; ERBB2_HUMAN; FAK1_HUMAN; FAK2_HUMAN; FGFR1_HUMAN; FGFR2_HUMAN; FGFR3_HUMAN; FGFR4_HUMAN; FLT3_HUMAN; FYN_HUMAN; GSK3A_HUMAN; GSK3B_HUMAN; HCK_HUMAN; IGF1R_HUMAN; IKKA_HUMAN; IKKE_HUMAN; INSR_HUMAN; ITK_HUMAN; JAK2_HUMAN; JAK3_HUMAN; KAPCA_HUMAN; KAPCB_HUMAN; KC1G2_HUMAN; KIT_HUMAN; KPCA_HUMAN; KPCB_HUMAN; KPCD1_HUMAN; KPCE_HUMAN; KPCT_HUMAN; KS6A5_HUMAN; KSYK_HUMAN; LCK_HUMAN; MAPK2_HUMAN; MET_HUMAN; MK01_HUMAN; MK03_HUMAN; MK10_HUMAN; MK12_HUMAN; MK14_HUMAN; MP2K1_HUMAN; MP2K2_HUMAN; MP2K4_HUMAN; NTRK1_HUMAN; PDPK1_HUMAN; PGFRA_HUMAN; PGFRB_HUMAN; PIM1_HUMAN; PK3CA_HUMAN; PLK1_HUMAN; Q9Y354_HUMAN; RET_HUMAN; RIPK1_HUMAN; SRC_HUMAN; STA5B_HUMAN; AURKA_HUMAN; TBA1A_HUMAN; TGFR2_HUMAN; TYRO3_HUMAN; VGFR1_HUMAN; VGFR2_HUMAN; VGFR3_HUMAN; WEE1_HUMAN; YES_HUMAN |
| Erlotinib | AKT1_HUMAN |
| Gefitinib | AKT1_HUMAN |
| Imatinib methansulfonate | AKT1_HUMAN; AAPK1_HUMAN; AAPK2_HUMAN; ABCG2_HUMAN; ABL1_HUMAN; ABL2_HUMAN; ABL_MLVAB; ACK1_HUMAN; ACV1B_HUMAN; ACVL1_HUMAN; ACVR1_HUMAN; ALBU_HUMAN; BCR_HUMAN; BTK_HUMAN; CAH12_HUMAN; CAH1_HUMAN; CAH2_HUMAN; CAH4_HUMAN; CAH9_HUMAN; CDK2_HUMAN; CDK5_HUMAN; CDK7_HUMAN; CDK8_HUMAN; CDK9_HUMAN; CLK1_HUMAN; CP1A2_HUMAN; CP2C8_HUMAN; CP2C9_HUMAN; CP2CJ_HUMAN; CP2D6_HUMAN; CP3A4_HUMAN; CP3A5_HUMAN; CP3A7_HUMAN; CSF1R_HUMAN; CSK21_HUMAN; CSK_HUMAN; DAPK1_HUMAN; DDR1_HUMAN; EGFR_HUMAN; EPHA2_HUMAN; EPHB1_HUMAN; ERBB2_HUMAN; ETV6_HUMAN; FAK1_HUMAN; FAK2_HUMAN; FGFR1_HUMAN; FGFR2_HUMAN; FGFR3_HUMAN; FGFR4_HUMAN; FLT3_HUMAN; FYN_HUMAN; GSK3A_HUMAN; GSK3B_HUMAN; HCK_HUMAN; HDAC1_HUMAN; HDAC6_HUMAN; IGF1R_HUMAN; IKKE_HUMAN; INSR_HUMAN; ITK_HUMAN; JAK2_HUMAN; JAK3_HUMAN; KAPCA_HUMAN; KAPCB_HUMAN; KC1G2_HUMAN; KIT_HUMAN; KPCA_HUMAN; KPCD1_HUMAN; KPCE_HUMAN; KPCT_HUMAN; KS6A5_HUMAN; KSYK_HUMAN; LCK_HUMAN; MAPK2_HUMAN; MDR1_HUMAN; MET_HUMAN; MK01_HUMAN; MK03_HUMAN; MK10_HUMAN; MK12_HUMAN; MK14_HUMAN; MP2K1_HUMAN; MP2K2_HUMAN; MP2K4_HUMAN; NTRK1_HUMAN; NU214_HUMAN; O43519_HUMAN; PDPK1_HUMAN; PGFRA_HUMAN; PGFRB_HUMAN; PIM1_HUMAN; PK3C3_HUMAN; PK3CA_HUMAN; PLK1_HUMAN; PRKDC_HUMAN; Q12843_HUMAN; Q12844_HUMAN; Q13688_HUMAN; Q13689_HUMAN; Q13691_HUMAN; Q13746_HUMAN; Q13914_HUMAN; Q13915_HUMAN; Q14708_HUMAN; Q14709_HUMAN; Q59F04_HUMAN; Q59F19_HUMAN; Q59FK4_HUMAN; KIT_HUMAN; PGFRA_HUMAN; Q6QJE4_HUMAN; Q7Z3C4_HUMAN; Q8TCG9_HUMAN; KIT_HUMAN; Q9P071_HUMAN; Q9UME7_HUMAN; RET_HUMAN; RIPK1_HUMAN; SRC_HUMAN; AURKA_HUMAN; TBA1A_HUMAN; TGFR2_HUMAN; TYRO3_HUMAN; VGFR1_HUMAN; VGFR2_HUMAN; VGFR3_HUMAN; WEE1_HUMAN; YES_HUMAN |
| Lapatinib ditosylate | AKT1_HUMAN |
| Simvastatin | AKT1_HUMAN |
| Sorafenibum | AKT1_HUMAN |
| Sunitinib | AKT1_HUMAN |
| Selumetinib | AKT1_HUMAN |
| Trametinib | AKT1_HUMAN |
| Rapamycin | AKT1_HUMAN; FKBP5_HUMAN; FKBP4_HUMAN; FKBP2_HUMAN; FKB1B_HUMAN; FKB1A_HUMAN; FKB1A_HUMAN; CP3A4_HUMAN; CP3A5_HUMAN; CP3A7_HUMAN; FKBP3_HUMAN; MTOR_HUMAN; MDR1_HUMAN; O60213_HUMAN; OPRK_HUMAN; MTOR_HUMAN; Q53GD8_HUMAN; Q53GX4_HUMAN; Q53XJ5_HUMAN; Q59GY9_HUMAN; FKBP5_HUMAN; Q5W0X3_HUMAN; Q6TBL4_HUMAN; Q6ZR21_HUMAN; Q96QW8_HUMAN |
| Atovaquone | PYRD_HUMAN; PPIA_HUMAN; PPIG_HUMAN |
| Essigsaeure | PYRD_HUMAN; PPIA_HUMAN; PPIG_HUMAN |
| Huanghuahaosu | PYRD_HUMAN; PPIA_HUMAN; PPIG_HUMAN |
| Hydroxycinchophene | PYRD_HUMAN; PPIA_HUMAN; PPIG_HUMAN |
| Leflunomide | PYRD_HUMAN; PPIA_HUMAN; PPIG_HUMAN |
| Athylenglykol | FKBP4_HUMAN |
| Methylsulfinylmethane | FKBP4_HUMAN |
| Carboxypyrrolidine | PPIB_HUMAN; PPIC_HUMAN; PPIH_HUMAN; PPIC_HUMAN; PPIH_HUMAN |
| Pimecrolimus | FKB1A_HUMAN |
| Tacarolimus | FKB1A_HUMAN |
| Thiabendazolum | FKB1A_HUMAN |
| Chloroquine | CP1A1_HUMAN; CP2C8_HUMAN; CP2D6_HUMAN; CP3A4_HUMAN; CP3A5_HUMAN; DHE3_HUMAN; DHE4_HUMAN; GSTA2_HUMAN; GSTA3_HUMAN; HBA_HUMAN; KCNH2_HUMAN; NQO2_HUMAN; PRIO_HUMAN; TLR9_HUMAN; YP_009725307.1 |
| Cyclosporine a | CP1A2_HUMAN; CP2C9_HUMAN; CP2CJ_HUMAN; CP2D6_HUMAN; CP3A4_HUMAN; MDR1_HUMAN; MRP2_HUMAN; PPIA_HUMAN |
| Saracatinib | ABL1_HUMAN; CSK_HUMAN; EGFR_HUMAN; KIT_HUMAN; LCK_HUMAN; SRC_HUMAN; VGFR2_HUMAN |
| Promethazine | 5HT2A_HUMAN; ACM1_HUMAN; ACM2_HUMAN; ACM3_HUMAN; ACM4_HUMAN; ACM5_HUMAN; ADA1A_HUMAN; CP2B6_HUMAN; CP2C9_HUMAN; CP2D6_HUMAN; DRD2_HUMAN; HRH1_HUMAN; PRIO_HUMAN; Q38LF9_HUMAN; Q38LG0_HUMAN; Q38LG2_HUMAN |
| Thiethylperazine | 5HT2A_HUMAN; 5HT2C_HUMAN; ACM1_HUMAN; ACM2_HUMAN; ACM3_HUMAN; ACM4_HUMAN; ACM5_HUMAN; ADA1A_HUMAN; DRD1_HUMAN; DRD2_HUMAN; DRD3_HUMAN; DRD4_HUMAN; DRD5_HUMAN |
| Fluphenazine | 5HT6R_HUMAN; CP1A2_HUMAN; CP2C8_HUMAN; CP2C9_HUMAN; CP2D6_HUMAN; CP2E1_HUMAN; DRD1_HUMAN; DRD2_HUMAN; DRD3_HUMAN; DRD4_HUMAN; DRD5_HUMAN; HRH1_HUMAN; HRH3_HUMAN |
| Triflupromazine | 5HT6R_HUMAN; CP1A2_HUMAN; CP2C8_HUMAN; CP2C9_HUMAN; CP2D6_HUMAN; CP2E1_HUMAN; DRD1_HUMAN; DRD2_HUMAN; DRD3_HUMAN; DRD4_HUMAN; DRD5_HUMAN; HRH1_HUMAN; HRH3_HUMAN |
| Chlorpromazine | 5HT1A_HUMAN; 5HT2A_HUMAN; 5HT6R_HUMAN; ALBU_HUMAN; CP1A2_HUMAN; CP2D6_HUMAN; CP2E1_HUMAN; CP3A4_HUMAN; DRD1_HUMAN; DRD2_HUMAN; DRD3_HUMAN; DRD5_HUMAN; HRH1_HUMAN; HRH3_HUMAN; KCNH2_HUMAN; MDR1_HUMAN; NMD3A_HUMAN; NMD3B_HUMAN; PRIO_HUMAN |
| Mycophenolic acid | ADRB2_HUMAN; IMDH1_HUMAN; IMDH2_HUMAN; PPARG_HUMAN; Q4R0Z5_HUMAN; Q4R0Z6_HUMAN; Q4R0Z7_HUMAN; Q53E75_HUMAN; Q5DSZ5_HUMAN; Q5DSZ6_HUMAN; Q5DSZ7_HUMAN; Q5DSZ8_HUMAN; Q5DSZ9_HUMAN; UD14_HUMAN; Q5DT01_HUMAN; Q5DT02_HUMAN; Q5DT03_HUMAN; Q5K632_HUMAN; Q5K672_HUMAN; Q5K673_HUMAN; Q5K674_HUMAN; Q5QTE2_HUMAN; Q75XT3_HUMAN; Q75XT5_HUMAN; UD110_HUMAN; Q8WUQ4_HUMAN; Q8WX89_HUMAN; Q8WX90_HUMAN; Q8WX91_HUMAN; Q96DH9_HUMAN; Q96PN9_HUMAN; Q96PP0_HUMAN; Q96PP1_HUMAN; Q9BX76_HUMAN; Q9H3F9_HUMAN; Q9H3G0_HUMAN; Q9H3G1_HUMAN; Q9H3G2_HUMAN; Q9UK62_HUMAN; UD110_HUMAN; UD11_HUMAN; UD13_HUMAN; UD14_HUMAN; UD15_HUMAN; UD16_HUMAN; UD17_HUMAN; UD18_HUMAN |
| Rocaglamide | NFKB1_HUMAN; TF65_HUMAN |
| Silvestrol | IF4A1_HUMAN; IF4A2_HUMAN; IF4A3_HUMAN |
| Favipiravir | RDRP_I56A0; YP_009725307.1 |
| Ribavirin | 5NTC_HUMAN; ADK_HUMAN; ENPP1_HUMAN; IMDH1_HUMAN; IMDH2_HUMAN; L_PI2HT; NTP1_MCV1; NPH2_MCV1; RDRP_I56A0; YP_009725307.1; K4LC41_9BETC; PLpro_SARS-CoV-2 |
| Penciclovir | DPOL_HHV11; KITH_HHV11; KITH_HHV1C; YP_009725307.1 |
| Disulfiram | AL1A1_HUMAN; ALDH2_HUMAN; AMD_HUMAN; CP1A2_HUMAN; CP2A6_HUMAN; CP2B6_HUMAN; CP2C8_HUMAN; CP2C9_HUMAN; CP2D6_HUMAN; CP2E1_HUMAN; CP3A4_HUMAN; CP3A5_HUMAN; DOPO_HUMAN; AMD_HUMAN; Q13749_HUMAN; Q16868_HUMAN; Q3YA63_HUMAN; Q4LBD0_HUMAN; Q59EJ0_HUMAN; Q5SYQ7_HUMAN; Q5SYQ8_HUMAN; Q5SYQ9_HUMAN; AL1A1_HUMAN; Q6NWT7_HUMAN; CP2E1_HUMAN; Q7KYY0_HUMAN; TRPA1_HUMAN; K4LC41_9BETC; PLpro_SARS-CoV-2 |
| Lopinavir | CP1A2_HUMAN; CP2B6_HUMAN; CP2C9_HUMAN; CP2CJ_HUMAN; CP2D6_HUMAN; CP3A4_HUMAN; MDR1_HUMAN; O90777_9PLVG; POL_HV1B1; Q9QM22_9HIV1; TFPI1_HUMAN; K4LC41_9BETC; PLpro_SARS-CoV-2; 6LU7:A; 6LU7:C |
| Ritonavir | CP1A2_HUMAN; CP2B6_HUMAN; CP2C8_HUMAN; CP2C9_HUMAN; CP2CJ_HUMAN; CP2D6_HUMAN; CP2E1_HUMAN; CP3A4_HUMAN; CP3A5_HUMAN; CP3A7_HUMAN; CP7A1_HUMAN; MDR1_HUMAN; O90777_9PLVG; POL_HV1H2; K4LC41_9BETC; PLpro_SARS-CoV-2; 6LU7:A; 6LU7:C |
| Darunavir | MDR1_HUMAN; O90777_9PLVG; POL_HV1B1; 6LU7:A; 6LU7:C |
| Nafamostat | C1R_HUMAN; C1S_HUMAN; CATD_HUMAN; FA10_HUMAN; MMP13_HUMAN; PLMN_HUMAN; THRB_HUMAN; TRY1_HUMAN; 6M0J:A; 6M0J:E; 6LZG:A; 6VSB:A; 6VXX:A |
| Resveratrol | ABL1_HUMAN; AKT1_HUMAN; CAH12_HUMAN; CAH1_HUMAN; CAH2_HUMAN; CAH4_HUMAN; CAH9_HUMAN; CDK4_HUMAN; CP1A1_HUMAN; CP1A2_HUMAN; CP1B1_HUMAN; CSK21_HUMAN; DCOR_HUMAN; ESR1_HUMAN; JUN_HUMAN; LCK_HUMAN; LKHA4_HUMAN; MK01_HUMAN; NQO2_HUMAN; PGH1_HUMAN; PGH2_HUMAN; PK3C3_HUMAN; PTN1_HUMAN; Q1HBJ4_HUMAN; Q3YA63_HUMAN; Q499G7_HUMAN; Q9Y354_HUMAN; TTHY_HUMAN |
| Gemcitabine hydrochloride | DCK_HUMAN; KCY_HUMAN; KITM_HUMAN; Q6I8R3_HUMAN; Q8IZR3_HUMAN; RIR1_HUMAN; TYSY_HUMAN |
| Amodiaquine | CP1A1_HUMAN; CP1A2_HUMAN; CP1B1_HUMAN; CP2C8_HUMAN; CP2C9_HUMAN; CP2CJ_HUMAN; CP2D6_HUMAN; CP3A4_HUMAN; HBA_HUMAN; HNMT_HUMAN; KCNH2_HUMAN |
| Mefloquine | CP19A_HUMAN; CP2D6_HUMAN; CP3A4_HUMAN; HBA_HUMAN |
| Loperamide | CAC1A_HUMAN; COLI_HUMAN; CP2B6_HUMAN; CP2C8_HUMAN; CP2D6_HUMAN; CP3A4_HUMAN; NMDE1_HUMAN; NMDE2_HUMAN; NMDE3_HUMAN; NMDE4_HUMAN; OPRD_HUMAN; OPRM_HUMAN; OPRX_HUMAN |
| Oseltamivir | NEUR1_HUMAN; NEUR2_HUMAN; NEUR3_HUMAN; NEUR4_HUMAN; NRAM_I83A1; NRAM_INBLE |
| Moexipril hydrochloride | ACE2_HUMAN |
| Imatinib mesylate | AAPK1_HUMAN; AAPK2_HUMAN; ABCG2_HUMAN; ABL1_HUMAN; ABL2_HUMAN; ABL_MLVAB; ACK1_HUMAN; ACV1B_HUMAN; ACVL1_HUMAN; ACVR1_HUMAN; AKT1_HUMAN; ALBU_HUMAN; BCR_HUMAN; BTK_HUMAN; CAH12_HUMAN; CAH1_HUMAN; CAH2_HUMAN; CAH4_HUMAN; CAH9_HUMAN; CDK2_HUMAN; CDK5_HUMAN; CDK7_HUMAN; CDK8_HUMAN; CDK9_HUMAN; CLK1_HUMAN; CP1A2_HUMAN; CP2C8_HUMAN; CP2C9_HUMAN; CP2CJ_HUMAN; CP2D6_HUMAN; CP3A4_HUMAN; CP3A5_HUMAN; CP3A7_HUMAN; CSF1R_HUMAN; CSK21_HUMAN; CSK_HUMAN; DAPK1_HUMAN; DDR1_HUMAN; EGFR_HUMAN; EPHA2_HUMAN; EPHB1_HUMAN; ERBB2_HUMAN; ETV6_HUMAN; FAK1_HUMAN; FAK2_HUMAN; FGFR1_HUMAN; FGFR2_HUMAN; FGFR3_HUMAN; FGFR4_HUMAN; FLT3_HUMAN; FYN_HUMAN; GSK3A_HUMAN; GSK3B_HUMAN; HCK_HUMAN; HDAC1_HUMAN; HDAC6_HUMAN; IGF1R_HUMAN; IKKE_HUMAN; INSR_HUMAN; ITK_HUMAN; JAK2_HUMAN; JAK3_HUMAN; KAPCA_HUMAN; KAPCB_HUMAN; KC1G2_HUMAN; KIT_HUMAN; KPCA_HUMAN; KPCD1_HUMAN; KPCE_HUMAN; KPCT_HUMAN; KS6A5_HUMAN; KSYK_HUMAN; LCK_HUMAN; MAPK2_HUMAN; MDR1_HUMAN; MET_HUMAN; MK01_HUMAN; MK03_HUMAN; MK10_HUMAN; MK12_HUMAN; MK14_HUMAN; MP2K1_HUMAN; MP2K2_HUMAN; MP2K4_HUMAN; NTRK1_HUMAN; NU214_HUMAN; O43519_HUMAN; PDPK1_HUMAN; PGFRA_HUMAN; PGFRB_HUMAN; PIM1_HUMAN; PK3C3_HUMAN; PK3CA_HUMAN; PLK1_HUMAN; PRKDC_HUMAN; Q12843_HUMAN; Q12844_HUMAN; Q13688_HUMAN; Q13689_HUMAN; Q13691_HUMAN; Q13746_HUMAN; Q13914_HUMAN; Q13915_HUMAN; Q14708_HUMAN; Q14709_HUMAN; Q59F04_HUMAN; Q59F19_HUMAN; Q59FK4_HUMAN; KIT_HUMAN; PGFRA_HUMAN; Q6QJE4_HUMAN; Q7Z3C4_HUMAN; Q8TCG9_HUMAN; KIT_HUMAN; Q9P071_HUMAN; Q9UME7_HUMAN; RET_HUMAN; RIPK1_HUMAN; SRC_HUMAN; AURKA_HUMAN; TBA1A_HUMAN; TGFR2_HUMAN; TYRO3_HUMAN; VGFR1_HUMAN; VGFR2_HUMAN; VGFR3_HUMAN; WEE1_HUMAN; YES_HUMAN |
| Remdesivir | YP_009725307.1 |
| Galidesivir | YP_009725307.1 |
| Valganciclovir | YP_009725307.1 |
| Chlorhexidine | YP_009725307.1; 6LU7:A; 6LU7:C |
| Ceftibuten | YP_009725307.1 |
| Fenoterol | YP_009725307.1 |
| Fludarabine | YP_009725307.1 |
| Itraconazole | YP_009725307.1 |
| Cefuroxime | YP_009725307.1 |
| Atovaquone | YP_009725307.1 |
| Chenodeoxycholic acid | YP_009725307.1 |
| Cromolyn | YP_009725307.1 |
| Pancuronium bromide | YP_009725307.1 |
| Cortisone | YP_009725307.1 |
| Tibolone | YP_009725307.1 |
| Novobiocin | YP_009725307.1 |
| Silybin | YP_009725307.1 |
| Idarubicin | YP_009725307.1 |
| Bromocriptine | YP_009725307.1 |
| Diphenoxylate | YP_009725307.1 |
| Benzylpenicilloyl g | YP_009725307.1 |
| Dabigatran etexilate | YP_009725307.1 |
| Amodiquine | YP_009725307.1 |
| 2-amino-9-[4-hydroxy-3-(hydroxymethyl)butyl]-3h-purin-6-one | YP_009725307.1 |
| Valganciclovir | K4LC41_9BETC; PLpro_SARS-CoV-2 |
| Beta-thymidine | K4LC41_9BETC; PLpro_SARS-CoV-2 |
| Riboflavin | K4LC41_9BETC; PLpro_SARS-CoV-2 |
| Reproterol | K4LC41_9BETC; PLpro_SARS-CoV-2 |
| Chloramphenicol | K4LC41_9BETC; PLpro_SARS-CoV-2 |
| Doxycycline | K4LC41_9BETC; PLpro_SARS-CoV-2; 6LU7:A; 6LU7:C |
| Iopromide | K4LC41_9BETC; PLpro_SARS-CoV-2 |
| Levodropropizine | K4LC41_9BETC; PLpro_SARS-CoV-2 |
| Floxuridine | K4LC41_9BETC; PLpro_SARS-CoV-2 |
| Tigecycline | K4LC41_9BETC; PLpro_SARS-CoV-2; 6LU7:A; 6LU7:C |
| Glutathione | K4LC41_9BETC; PLpro_SARS-CoV-2 |
| Ademetionine | K4LC41_9BETC; PLpro_SARS-CoV-2 |
| Masoprocol | K4LC41_9BETC; PLpro_SARS-CoV-2 |
| Isotretinoin | K4LC41_9BETC; PLpro_SARS-CoV-2 |
| Dantrolene | K4LC41_9BETC; PLpro_SARS-CoV-2 |
| Sulfasalazine | K4LC41_9BETC; PLpro_SARS-CoV-2 |
| Cobicistat | 6LU7:A; 6LU7:C |
| Nelfinavir | 6LU7:A; 6LU7:C |
| Indinavir | 6LU7:A; 6LU7:C |
| Atazanavir | 6LU7:A; 6LU7:C |
| Saquinavir | 6LU7:A; 6LU7:C |
| Tipranavir | 6LU7:A; 6LU7:C |
| Amprenavir | 6LU7:A; 6LU7:C |
| Fosamprenavir | 6LU7:A; 6LU7:C |
| Lymecycline | 6LU7:A; 6LU7:C |
| Alfuzosin | 6LU7:A; 6LU7:C |
| Cilastatin | 6LU7:A; 6LU7:C |
| Famotidine | 6LU7:A; 6LU7:C |
| Almitrine | 6LU7:A; 6LU7:C |
| Progabide | 6LU7:A; 6LU7:C |
| Nepafenac | 6LU7:A; 6LU7:C |
| Carvedilol | 6LU7:A; 6LU7:C |
| Demeclocycline | 6LU7:A; 6LU7:C |
| Montelukast | 6LU7:A; 6LU7:C |
| Mimosine | 6LU7:A; 6LU7:C |
| Flavin mononucleotide | 6LU7:A; 6LU7:C |
| Lutein | 6LU7:A; 6LU7:C |
| Cefpiramide | 6LU7:A; 6LU7:C |
| Phenethicillin | 6LU7:A; 6LU7:C |
| Candoxatril | 6LU7:A; 6LU7:C |
| Nicardipine | 6LU7:A; 6LU7:C |
| Estradiol valerate | 6LU7:A; 6LU7:C |
| Pioglitazone | 6LU7:A; 6LU7:C |
| Conivaptan | 6LU7:A; 6LU7:C |
| Telmisartan | 6LU7:A; 6LU7:C |
| Oxytetracycline | 6LU7:A; 6LU7:C |
| Lmt-28 | IL6RB_HUMAN |
