# Supplementary material for: Virtual screening of Indonesian herbal compounds as COVID-19 supportive therapy: machine learning and pharmacophore modeling approaches
Source: BMC Complement Med Ther. 2022 Aug 3;22:207. doi: 10.1186/s12906-022-03686-y (PMC9347098; doi:10.1186/s12906-022-03686-y)
Supplement: Supplementary file 4 — Additional file 4. Training and test dataset for ligand-based method. [file 12906_2022_3686_MOESM4_ESM.docx]

**Additional file 4.** Training and test dataset for Ligand-based methods

Table 1. Training set

| **Number** | **Compounds** |
| --- | --- |
| 1 | Atazanafir |
| 2 | Cobicistat |
| 3 | Darunavir |
| 4 | Favipiravir |
| 5 | Galidesivir |
| 6 | Indinavir |
| 7 | Lopinavir |
| 8 | Nafamostat |
| 9 | Oseltamivir |
| 10 | Remdesivir |
| 11 | Ribavirin |
| 12 | Ritonavir |
| 13 | Saquinavir |
| 14 | Umifenovir |
| 15 | ASC09F^1^ |

Table 2. Test set

| **Number** | **Compounds** | **Number** | **Compounds** |
| --- | --- | --- | --- |
| 1 | Abacavir | 16 | SSYA10-001^1^ |
| 2 | Bortezomib | 17 | PX-12^1^ |
| 3 | Carfilzomib | 18 | GC813^1^ |
| 4 | Elvitegravir | 19 | GC376^1^ |
| 5 | Enzaplatovir | 20 | 6'-Fluorinatedaristeromycin  Analogues (Compound 2c)^1^ |
| 6 | Fosamprenavir | 21 | Acyclovir fleximer  Analogues (Compound 2)^1^ |
| 7 | Maribavir | 22 | Montelukast |
| 8 | Penciclovir | 23 | Deoxyrhubarb |
| 9 | Presatovir | 24 | Polydatin |
| 10 | Raktegravir | 25 | Compound 21^2^ |
| 11 | Resveratrol | 26 | Compound 22^2^ |
| 12 | Silvestrol | 27 | Compound 23^2^ |
| 13 | Tideglusib | 28 | Compound 24^2^ |
| 14 | Tipranavir | 29 | Compound 25^2^ |
| 15 | TDZD-8^1^ | 30 | Cinanserin |

Reference

1. Li G, De Clercq E. Therapeutic options for the 2019 novel coronavirus (2019-nCoV). Nat Rev Drug Discov. 2020;19:149–50.
2. The joint research team of ShanghaiTech and Shanghai Institute of Materia Medica Discovers a Series of Drugs with Potential for COVID-19 Treatment. 2020. <http://www.shanghaitech.edu.cn/eng/2020/0215/c1260a50244/page.htm>. Accessed 20 February 2020.
