# Supplementary material for: Virtual screening of Indonesian herbal compounds as COVID-19 supportive therapy: machine learning and pharmacophore modeling approaches
Source: BMC Complement Med Ther. 2022 Aug 3;22:207. doi: 10.1186/s12906-022-03686-y (PMC9347098; doi:10.1186/s12906-022-03686-y)
Supplement: Supplementary file 5 — Additional file 5. The optimal hyper-parameter values of each model. [file 12906_2022_3686_MOESM5_ESM.docx]

**Additional File 5**  The Optimal hyper-parameter values of each model

| **Method** | **Hyper-parameter** | **Value** |
| --- | --- | --- |
| Multilayer Perceptron (MLP) | Activation function | Relu |
|  | Alpha | 0.5 |
|  | Hidden layer size | (640,320) |
|  | Learning rate | Adaptive |
|  | Batch size | 256 |
|  | Optimizer | Adam |
|  | Class weight | ‘balanced’ |
| Random Forest (RF) | Number of tree | 1000 |
|  | Minimum samples split | 2 |
|  | mtry | 1281 |
|  | bootstrap | True |
|  | Max sample | 10000 |
|  | Class weight | ‘balanced’ |
| Support Vector Machine (SVM) | Kernel | RBF |
|  | C | 2.0 |
|  | Gamma | 0.5 |
|  | Class weight | ‘balanced’ |
