# Supplementary material for: Virtual screening of Indonesian herbal compounds as COVID-19 supportive therapy: machine learning and pharmacophore modeling approaches
Source: BMC Complement Med Ther. 2022 Aug 3;22:207. doi: 10.1186/s12906-022-03686-y (PMC9347098; doi:10.1186/s12906-022-03686-y)
Supplement: Supplementary file 6 — Additional file 6. The prediction results of herbal compounds. [file 12906_2022_3686_MOESM6_ESM.docx]

**Additional File 6** The prediction results of herbal compound

| **No** | **Protein Target** | **Herbal Compound** |
| --- | --- | --- |
| 1. | 3CLPro | Amaranthine, Methylthio, arabinopyrano, Peonidin-3, Quercetin-3, Sinigrin, **Hesperidine**, Myricetin-3, (+)-2,3-Dihydro-9-hydroxy, Cyanidin-3, Scutellarein, Spiraeoside, Glucoputran, Isoforskolin, Kaempferol-3, Rhamnetin, Glucosinolate, ß-pinene, ß-caryophyllene, A-thujene, Y-mangostin, Viridiflorene, Vicenin, Ursolic acid, Tinocordiside, Tetradecanol, Terpinyl acetate, Tartaric acid, Stigmasterol-3-o-glucoside, Sterculic acid, Stearic acid, Spathulenol, Sabinene, Rutin, Ruberythric acid, Ricinoleic acid, Quercitrin, Quercetin-3-o-rhamnoside, Propionic acid, Piperitone, Petunidin-3-gentiobioside, Pelargonic acid, Palmitic acid, Oleanolic acid, Nonanal, Nirurin, Nerolidol, Nerol/geraniol, Naringin, Myristic acid, Morindin, Momordol, Momordicine 1, Momordicilin, Momordenol, Mannose-6-phosphate, Mangiferin, Malvidin-3-laminaribioside, Malvalic acid, Malic acid, Linalool, Andrographolide type 1, Andrographatoside, Andrograpanin, 14-deoxy-11-oxoandrographolide, 14-deoxy-11,12-didehydroandrographolide 2, 14-deoxy-11,12-didehydroandrographolide, Caprylic acid, Cyanidin 3-glucoside, Cyanidin 3-xyloglucoside, Cyanidin-3-sambubioside, Delphinidin 3-sambubioside, Delphinidin-3-glucoside, Delphinin, Glycolic acid, Gossypetin 3-glucoside, Hibiscus acid, Blumenol a, Blumenol b, Ligan library pare, 24-isopropyl cholesterol, Amarulone, Astragalin, Beta sitosterol, Ellagic acid, Isoquercetrin, Caproic acid, Ethyl caproate, Ethyl caprylate, Kaempferol 3-o-alpha-l-rhamnopyranoside/afzelin, (3beta,23e)-3-hydroxy-27-norcycloart-23-en-25-one, Arabinose, B-sitosterol, Campesterol, Gychirrethinic acid, 1-hexanol, 2-heptadecanone, 3-hexen-1-ol, A-cadinol, Aromadendrene, Borneol, Bornyl acetate, B-salinene, Camphene, Caryophyllene oxide, Cis-linalool oxide (pyranoid), Epi-a-bisabolol, Farnesol, Fenchone, Geranial/citral, Geraniol, Germacrene-d, Heptan-2-one, Heptanal, Hexadecanol, Ledol, Betulinic acid, Cyanidin diglycoside, Delphinidin-3-gentiobioside, Epi-friedelanol, Friedelin, Hyperoside, Isoquercetin, Ligan library brotowali, Amritoside, Berberine2, Columbin, Cycloeucalenol, Cycloeucalenone, Goyaglycoside a, Galacturonic acid, Erythrodiol, Diosgenin, Cycloarthenol, Cucurbitins, Cucurbitane, Cucurbitacin r, Cucurbitacin d, Charine, Charantin, Andrographolide, Andrographolide type 4, Andrographolide type 3, Andrographolide type 2, 14-deoxy-11,12-didehydroandrographolide2, 11-hydroxymutakone, |
| 2. | PLPro | Methylthio, Sinigrin, Glucoputran, Glucosinolate, ß-pinene, ß-caryophyllene, A-thujene, A-terpinene, A-pinene, Y-mangostin, Ylangene, Y-cardinene, Viridiflorene, Valencene, Ursolic acid, Tinocordiside, Thymoquinone, Tetradecanol, Terpinyl acetate, Terpinolene, Tartaric acid, Stigmasterol-3-o-glucoside, Sterculic acid, Stearic acid, Spathulenol, Sinamaldehid, Safrole, Sabinene, Ricinoleic acid, Pyrocathecol, Propionic acid, Piperitone, Phellandre, Pelargonic acid, P-cymene, P-coumaric acid, Palmitoleic acid, Palmitic acid, Oxalic acid, Oleic acid, Oleanolic acid, Nonanal, N-heptadecane, Nerolidol, Nerol/geraniol, Myristic acid, Myrcene, Momordol, Momordicine 1, Momordenol, Methyl isoeugenol, Methyl eugenol, Methyl cinnamate, Methyl chavicol, Mannose-6-phosphate, Malvalic acid, Malic acid, Maleic acid, Linoleic acid, Linalool, Linalool acetate, Limonene, Andrograpanin, 14-deoxy-11,12-didehydroandrographolide 2, Anisaldehyde, Ascorbic acid, Caprylic acid, Citric acid, Formic acid, Glycolic acid, Hibiscus acid, Blumenol a, Blumenol b, Ligan library pare, 24-isopropyl cholesterol, Beta sitosterol, Estradiol, Caproic acid, Ethyl caproate, Ethyl caprylate, (3beta,23e)-3-hydroxy-27-norcycloart-23-en-25-one, Arabinose, B-sitosterol, Campesterol, Cinnamic acid, (e)-beta-farnesene, Z-beta-ocimene, (z)-cinnamyl acetate, 1,8-cineole, 1-hexanol, 2-heptadecanone, 3-carene, 3-hexen-1-ol, A-bergamoten, A-cadinene, A-cadinol, A-calacorene, Acetyl eugenol, A-copaene, A-humulene, A-muurolene, Aromadendrene, A-selinene, Benzaldehyde, Benzyl alcohol, Beta-ocimene, Borneol, Bornyl acetate, B-salinene, Cadalene, Camphene, Caryophyllene oxide, Cinnamaldehyde, Cinnamyl acetate, Cinnamyl alcohol, Cis calamenene, Cis-linalool oxide (pyranoid), Cuminaldehyde, D-cadinene, Epi-a-bisabolol, Ethyl cinnamate, Eugenyl acetate, Farnesol, Fenchone, Geranial/citral, Geraniol, Germacrene-d, Heptan-2-one, Heptanal, Hexadecanol, Hydrocinnamaldehyde, Hydrocinnamic acid, Isoeugenol, Ledol, Z-cinnamaldehyde, Betulinic acid, Epi-friedelanol, Friedelin, Ligan library brotowali, Berberine2, Cycloeucalenol, Cycloeucalenone, Goyaglycoside c, Goyaglycoside a, Galacturonic acid, Erythrodiol, Elaeostearic acid, Diosgenin, Cycloarthenol, Cucurbitane, Cryptoxanthin, Charantin, 14-deoxy-11,12-didehydroandrographolide2, 11-hydroxymutakone, |
| 3. | RdRp | Methylthio, arabinopyrano, Peonidin-3, Quercetin-3, Theviridoside, Sinigrin, Hesperidine, Myricetin-3, (+)-2,3-Dihydro-9-hydroxy, Cyanidin-3, Catalpol, Scandoside, Scutellarein, Spiraeoside, Geniposide, Oleoside, Majoroside, Glucoputran, Isoforskolin, Kaempferol-3, Rhamnetin, Psoralen, Glucosinolate, ß-pinene, ß-caryophyllene, A-thujene, A-terpinene, A-pinene, Y-mangostin, Ylangene, Y-cardinene, Viridiflorene, Vicenin, Valencene, Ursolic acid, Tinosporaside, Tinocordiside, Thymoquinone, Tetradecanol, Terpinyl acetate, Terpinolene, Tartaric acid, Stigmasterol-3-o-glucoside, Sterculic acid, Stearic acid, Spathulenol, Sinamaldehid, Sabinene, Rutin, Ruberythric acid, Ricinoleic acid, Quercitrin, Quercetin-3-o-rhamnoside, Propionic acid, Piperitone, Phellandre, Petunidin-3-gentiobioside, Pelargonic acid, P-cymene, Palmitoleic acid, Palmitic acid, Oxalic acid, Oleic acid, Oleanolic acid, Nonanal, Nirurin, N-heptadecane, Nerolidol, Nerol/geraniol, Neoandrographolid, Naringin, Myristic acid, Myrcene, Morindin, Momordol, Momordin i, Momordin lc, Momordin ib, Momordicinin, Momordicine 1, Momordicilin, Momordenol, Methyl cinnamate, Mannose-6-phosphate, Mangiferin, Malvidin-3-laminaribioside, Malvalic acid, Malic acid, Maleic acid, Linoleic acid, Linalool, Linalool acetate, Limonene, Andrographolide type 1, Andrographoside, Andrographatoside, Andrograpanin, 14-deoxyandrographoside, 14-deoxy-11-oxoandrographolide, 14-deoxy-11,12-didehydroandrographolide 2, 14-deoxy-11,12-didehydroandrographolide, Ascorbic acid, Caprylic acid, Citric acid, Cyanidin 3-glucoside, Cyanidin 3-xyloglucoside, Cyanidin-3-sambubioside, Delphinidin 3-sambubioside, Delphinidin-3-glucoside, Delphinin, Formic acid, Glycolic acid, Gossypetin 3-glucoside, Hibiscus acid, Blumenol a, Blumenol b, Ligan library pare, 24-isopropyl cholesterol, Amarulone, Astragalin, Beta sitosterol, Isoquercetrin, Asperuloside tetraacetate, Asperulosidic acid, Aucubin, Caproic acid, Ethyl caproate, Ethyl caprylate, Kaempferol 3-o-alpha-l-rhamnopyranoside/afzelin, (3beta,23e)-3-hydroxy-27-norcycloart-23-en-25-one, Accemannan, Aloinoside b, Arabinose, B-sitosterol, Campesterol, Cinnamic acid, Gibberelins, Gychirrethinic acid, (e)-beta-farnesene, Z-beta-ocimene, (z)-cinnamyl acetate, 1,8-cineole, 1-hexanol, 2-heptadecanone, 3-carene, 3-hexen-1-ol, A-bergamoten, A-cadinene, A-cadinol, A-calacorene, A-copaene, A-humulene, A-muurolene, Aromadendrene, A-selinene, Benzaldehyde, Benzyl alcohol, Benzyl benzoate, Beta-ocimene, Borneol, Bornyl acetate, B-salinene, Cadalene, Camphene, Caryophyllene oxide, Cinnamaldehyde, Cinnamyl acetate, Cinnamyl alcohol, Cis calamenene, Cis-linalool oxide (pyranoid), D-cadinene, Epi-a-bisabolol, Ethyl cinnamate, Farnesol, Fenchone, Geranial/citral, Geraniol, Germacrene-d, Heptan-2-one, Heptanal, Hexadecanol, Ledol, Z-cinnamaldehyde, Betulinic acid, Cyanidin diglycoside, Delphinidin-3-gentiobioside, Epi-friedelanol, Friedelin, Hyperoside, Isoquercetin, Ligan library brotowali, Amritoside, Berberine2, Columbin, Cycloeucalenol, Cycloeucalenone, Goyaglycoside h, Goyaglycoside g, Goyaglycoside f, Goyaglycoside e, Goyaglycoside c, Goyaglycoside a, Galacturonic acid, Erythrodiol, Elaeostearic acid, Diosgenin, Cycloarthenol, Cucurbitane, Cucurbitacin s, Cucurbitacin r, Cucurbitacin q, Cucurbitacin p, Cucurbitacin o, Cucurbitacin l, Cucurbitacin k, Cucurbitacin j, Cucurbitacin i, Cucurbitacin g/h, Cucurbitacin f, Cucurbitacin e, Cucurbitacin d, Cucurbitacin c, Cucurbitacin b, Cucurbitacin a, Cryptoxanthin, Charine, Charantin, Andrographolide, Andrographolide type 4, Andrographolide type 3, Andrographolide type 2, 14-deoxy-11,12-didehydroandrographolide2, 11-hydroxymutakone, |
