# Supplementary material for: Virtual screening of Indonesian herbal compounds as COVID-19 supportive therapy: machine learning and pharmacophore modeling approaches
Source: BMC Complement Med Ther. 2022 Aug 3;22:207. doi: 10.1186/s12906-022-03686-y (PMC9347098; doi:10.1186/s12906-022-03686-y)
Supplement: Supplementary file 7 — Additional file 7. Redocking result for 6LU7 native ligand. [file 12906_2022_3686_MOESM7_ESM.docx]

**Additional File 7** 6LU7’s native ligand redocking result


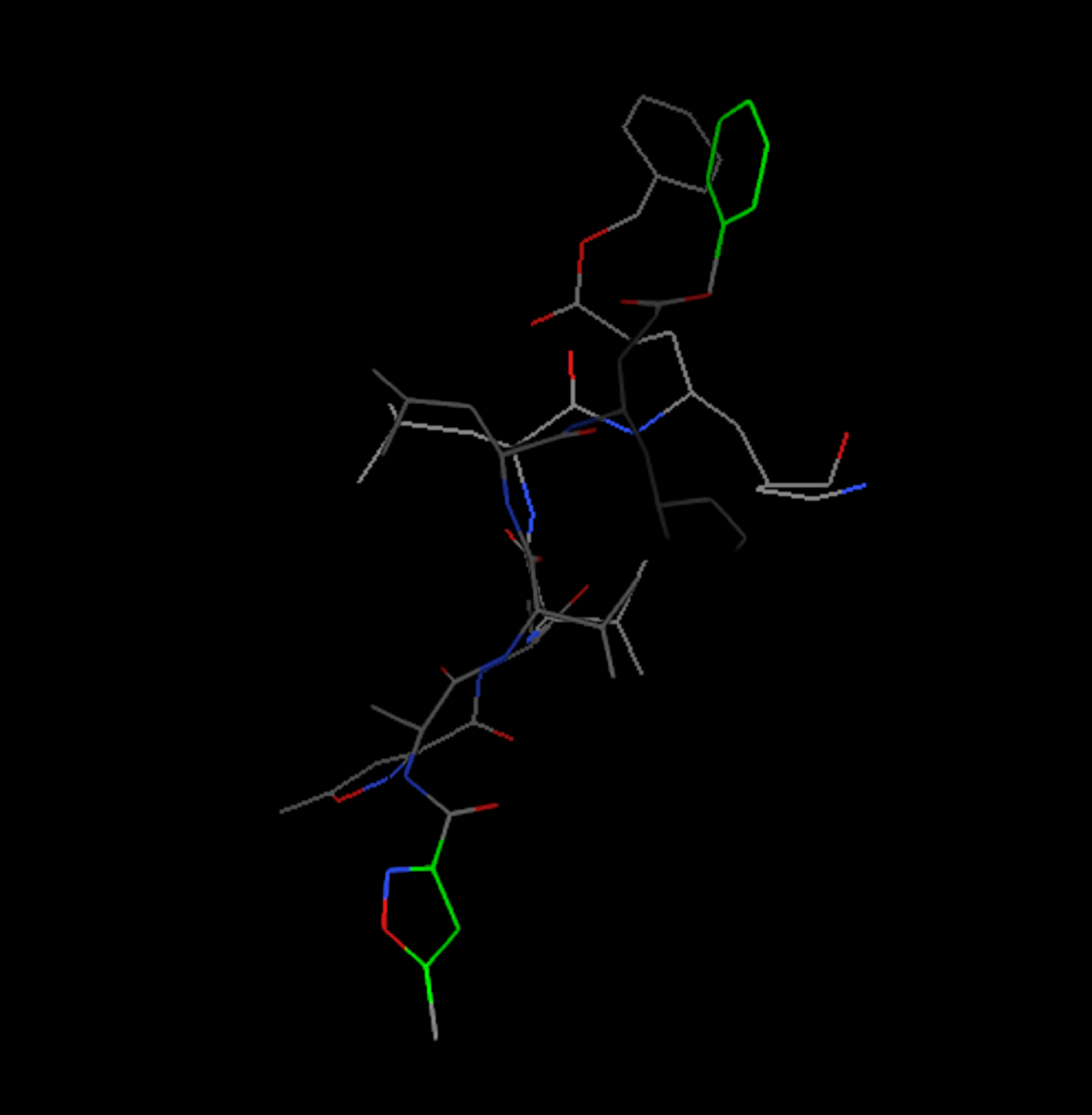


Coordinates of Central Grid Point of Maps = (-9.732, 11.403, 68.925) ; 40x56x40

Estimated Free Energy of Binding = -7.96 kcal/mol

RMSD = 1.78 A
